# Development of super-infective ternary vector systems for enhancing the *Agrobacterium*-mediated plant transformation and genome editing efficiency

**DOI:** 10.1093/hr/uhae187

**Published:** 2024-07-10

**Authors:** Jin-hee Jeong, Eun-young Jeon, Min Ki Hwang, Young Jong Song, Jae-Yean Kim

**Affiliations:** Nulla Bio Inc., 501 Jinjudaero, Jinju 660-701, Republic of Korea; Division of Applied Life Science (BK21 Four program), Plant Molecular Biology and Biotechnology Research Center, Gyeongsang National University, Jinju 660-701, Republic of Korea; Nulla Bio Inc., 501 Jinjudaero, Jinju 660-701, Republic of Korea; Division of Life Science, Gyeongsang National University, 501 Jinju-daero, Jinju 52828, Republic of Korea; Division of Applied Life Science (BK21 Four program), Plant Molecular Biology and Biotechnology Research Center, Gyeongsang National University, Jinju 660-701, Republic of Korea; Division of Applied Life Science (BK21 Four program), Plant Molecular Biology and Biotechnology Research Center, Gyeongsang National University, Jinju 660-701, Republic of Korea; Nulla Bio Inc., 501 Jinjudaero, Jinju 660-701, Republic of Korea; Division of Applied Life Science (BK21 Four program), Plant Molecular Biology and Biotechnology Research Center, Gyeongsang National University, Jinju 660-701, Republic of Korea; Division of Life Science, Gyeongsang National University, 501 Jinju-daero, Jinju 52828, Republic of Korea

## Abstract

*Agrobacterium*-mediated transformation remains a cornerstone of plant biology, fueling advancements in molecular genetics, new genomic techniques (NGTs), and the biotech industry. However, recalcitrant crops and technical hurdles persist as bottlenecks. The goal was to develop super-infective ternary vector systems that integrate a novel salicylic acid-degrading enzyme, GABA, and ethylene-degrading enzymes, targeting the transformation of crops by neutralizing plant defense system on *Agrobacterium*. Firstly, both the effect and activity of introducing enzymes were validated in EHA105, an important *Agrobacterium* strain. Our study demonstrates that all ternary vector (Tv) system variants significantly enhance reporter expression in transient assays with *Nicotiana benthamiana* and *Cannabis sativa*. Specifically, incorporating a constitutive virG mutation with novel enzyme combinations increased GFP and RUBY expression in *C. sativa* by >5-fold and 13-fold, respectively. The Tv system, combined with a geminivirus replicon, markedly boosted GUS gene expression in tomato, enhancing genome editing efficiency. Notably, compared to controls, Tv-VS demonstrated up to 18-fold and 4.5-fold increases in genome editing efficiency in *C. sativa* and tomato, respectively. Additionally, stable transformation rates in tomato and *Arabidopsis* improved significantly, with Tv-VS showing a remarkable 2.5-fold increase in transformation efficiency compared to control strains. The research marks notable progress in *Agrobacterium*-mediated plant transformation. The innovative ternary vectors overcome plant defense mechanisms, enabling genetic manipulation in previously challenging plant species. This development is anticipated to broaden the applications of plant genetic engineering, contributing to advancements in crop genome editing.

## Introduction

Over the past 100 years, the importance of *Agrobacterium* in plant biotechnology has become evident. Since the discovery of the bacterium causing crown gall disease, initially named *Bacterium tumefaciens* [1], it was subsequently named *Agrobacterium tumefaciens* [2]. Although some *Agrobacterium* species are non-pathogenic, the majority can induce tumors in various plants [3]. Initially, it was found that crown gall formation is associated with a large plasmid [4], and it has been observed that *Agrobacterium* can transfer genetic material to a wide range of plants, including most dicots and some monocots [4]. By this time, scientists had discovered the ability to replace oncogenes in T-DNA with genes of interest, marking the beginning of a new era in plant biotechnology with the tremendous potential for transferring genetic materials from bacteria to plants [5].

The previous study has found that *Agrobacterium* requires plant wounding or stomatal openings for invasion [6]. It has been further elucidated that plant wounding triggers the secretion of specific chemical compounds, including phenolic compounds, sugars, and certain amino acids, which are known to enhance *Agrobacterium*'s infection and pathogenic capabilities [7]. These compounds serve as signals that *Agrobacterium* detects, activating its virulence genes [8]. However, it is also important to acknowledge that not all responses to wounding facilitate infection; these plants secrete defensive compounds that can inhibit bacterial growth or infection.

The initiation of this mechanism involves a receptor protein VirA, which, upon detecting phenolic signals, activates VirG, a response regulator, by a phospho-relay system. The activated, phosphorylated VirG then operates as a transcription factor, binding to promoters of other *Vir* genes [9]. The Chromosomal Virulence (Chv) E protein, a periplasmic receptor, also facilitates sugar and acidic signal mediation with VirA [10, 11]. The ChvG/ChvI two-component system further plays a crucial role in activating additional *Vir* genes [12, 13], enhancing the *Agrobacterium*'s ability to interact with and transform plant cells. For natural transformation to occur, virulent *Agrobacterium* strains utilize its Ti-plasmid, crucial for integrating genetic material into the plant genome, to induce plant transformation. This process involves transferring the Ti-plasmid's T-DNA into the plant chromosome, facilitated by numerous Vir proteins [14]. The large Ti-plasmid was engineered into a binary system, comprising a disarmed helper plasmid and a binary vector with a T-DNA [15], facilitating gene cloning and plant biotechnology applications. However, the efficiency of *Agrobacterium*-mediated transformation varies, with many monocots and some dicots showing resistance [16, 17].

Interestingly, from the view of host plants, bioprocesses of *Agrobacterium*–plant interactions are an invasion. In amplified fragment length polymorphism (AFLP)-based transcript profiling, plant defense-related genes were identified in *Agrobacterium* infection [18]. Besides, *Agrobacterium*-infected plants have been reported to mount the plant innate immune response, such as MAPK activation, pattern-triggered immunity, phytohormone modulation, and reactive oxygen species (ROS) production [19–23]. Thus, the plants can launch a variety of immune responses and limit pathogen infection in *Agrobacterium–*plant interactions [24]. In nature, phytohormones are essential for the plant development process and pivotal in immune response [25]. Among them, salicylic acid (SA), indole-3-acetic acid (IAA), and ethylene in *Arabidopsis thaliana* were strongly accumulated after *Agrobacterium* infection. [26]. Notably, IAA acts as a competitor for inducer of VirA/VirG two-component system [27], and stimulates the synthesis of high amounts of ethylene [28–30]. Ethylene also influences *Agrobacterium*, and its interaction with plants induces tumor development through vascularization and epidermal disruption [31]. Moreover, previous studies have shown that ethylene is involved in the inhibition of *Agrobacterium*-mediated transformation in tomato, melon, and bottle gourd [32–35]. In addition to the universal phytohormones, plants contain another negative factor, such as γ-aminobutyric acid (GABA), 2,4-dihydroxy-7-methoxy-2H-1,4-benzoxazin-3(4H)-one (DIMBOA), and 2-hydroxy-4,7-dimethoxybenzoxazin-3-one (MDIBOA). Despite the presence of virulence signals, DIMBOA and MDIBOA shut down *Vir* gene expression and limit *Agrobacterium* growth in maize seedlings [36, 37]*.* Besides, GABA, non-protein amino acids, accumulates rapidly in response to biotic and abiotic stresses including *Agrobacterium* invasion and wounding, and plays a role in defense response [38–40]. In *Agrobacterium*-plant interaction, GABA also activates the *attKLM* operon, thereby degradation of the quorum-sensing (QS) signal [41]. Because high levels of QS signals are known to trigger a defense response in eukaryotic hosts, it was proposed that suppressing QS during the early stages of infection advantages *Agrobacterium* virulence [42, 43]. Taken together, *Agrobacterium* virulence is tightly modulated by phytohormone and plant-derived chemicals.

*Agrobacterium*'s properties for improving transformation are routinely exploited to engineer plants in the scope of fundamental genetic research. With a stepwise strategy to reach this aim, exogenous supplies of *Vir* gene inducer, such as acetosyringone (AS), sugar, and acidic condition, and suppressor of negative effectors, such as aminoethoxyvinylglycine (AVG), AgNO3, and silver thiosulfate (STS) are used for enhancing the efficiency of *Agrobacterium*-mediated plant transformation. From another viewpoint, *Agrobacterium* improvement might also show possibilities for achieving improved transformation efficiency [8, 32, 44]. One of the best ways to enhance transformation efficiencies is to use strains that overexpress specific *Vir* genes. Indeed, constitutive *virG* mutants, such as *virG*^I77V^, *virG*^N54D^, and *virG*^I106L^, dramatically improve transformation efficiencies in a variety of plants [45, 46]. Moreover, an increase of *VirG* wild-type copies increased transient callus transformation of celery (*Apium graveolens*) and rice [47]. VirA constitutive variants also achieve a high level of *Vir* gene expression without AS [48]. Although various elements to increase the expression of virulence genes have been studied, issues such as vector complexity and size have made their application difficult. In 2000, the introduction of *virG*^N54D^ into a third plasmid overcame these challenges and demonstrated the potential to upgrade *Agrobacterium* [49]. Recent advancements in deploying ternary vectors enriched with additional Virulence (Vir) genes not only heightened the efficiency of genetic transformation in maize but also significantly boosted the effectiveness of gene editing mediated by CRISPR/Cas9 systems [50–52]. Alternatively, *Agrobacterium* strains can be engineered to overcome the negative impact of plant-derived molecules. For instance, several versions of 'Super-*Agrobacterium* have been developed by introducing the GABA transaminase (*GabT*) or ACC deaminase (*AcdS*) gene in a ternary vector, so that it could break down GABA or ethylene or both on its own. These engineered strains showed the enhanced ability of T-DNA transfer in *Cucumis melo*, *Erianthus ravennae, Solanum lycopersicum,* and *Solanum torvum* [53–55].

As one of the phytohormones in plant defense, SA plays a regulatory role in response to pathogenic invasion [56–59]. Specifically, SA promotes the expression of *attKLM* operon, thereby activating the *Agrobacterium* quorum-quenching system. Furthermore, SA is directly implicated in the suppression of *Vir* genes through VirA/VirG two-component system [60, 61]. Indeed, SA was accumulated at the infected area in tobacco, and these amounts of SA affected negatively Vir regulon induction [61, 62]. *Pseudomonas putida* NahG gene was firstly introduced into *Arabidopsis* and tobacco plants so that SA-degrading NahG plants were shown to enhance susceptibility to diverse bacterial pathogens [63, 64]. As a salicylate hydroxylase, we hypothesize that NahG activity in *Agrobacterium* infection might play a role in the degradation of plant-secreted SA to enhance susceptibility to *Agrobacterium*. In this study, we postulated that the Tv system, equipped with an SA modulator, could be utilized to enhance an improved *Agrobacterium* platform for genetic transformation tailored to the specific characteristics of plants. Consequently, we developed versatile Tv systems to facilitate *Agrobacterium*-mediated plant transformation. To demonstrate the Tv system as a powerful technology, we applied it using several reporters, a geminiviral replicon system, and even a CRISPR/Cas9 system in tomato, *N. benthamiana*, *A. thaliana,* and *C. sativa*.

**Table 1:** List of ternary plasmids and binary plasmids that are used in this study

**Vector**	**Construct**	**Backbone plsamid**	**Description**
**Ternary vector**	**pRiA4-VIR**	Addgene 138 193 (Zhang, 2019)	pRiA4*-vir* gene cluster, a *vir* region derived from pTiBo542
	**Tv-GE**	pBBR1 (broad-host range shuttle vector)	pBBR1-*GabT/AcdS,* GABA, and ethylene-neutralizing elements
	**Tv-GEV**	pBBR1 (broad-host range shuttle vector)	pBBR1-*GabT*/*AcdS*/*virG*^N54D^, GABA, and ethylene-neutralizing elements with the expession of constitutively active VirG^N54D^
	**Tv-S**	pBBR1 (broad-host range shuttle vector)	pBBR1-*NahG,* SA-neutralizing element
	**Tv-GES**	pBBR1 (broad-host range shuttle vector)	pBBR1-*GabT*/*AcdS*/*NahG,* GABA, ethylene, and SA-neutralizing elements
	**Tv-VS**	pBBR1 (broad-host range shuttle vector)	pBBR1-*virG*^N54D^/*NahG,* SA-neutralizing elements the expession of constitutively active VirG^N54D^
	**Tv-GEVS**	pBBR1 (broad-host range shuttle vector)	pBBR1-*GabT*/*AcdS*/*virG*^N54D^/*NahG,* GABA, ethylene, and SA-neutralizing elements the expession of constitutively active VirG^N54D^
**Conventional binary vector**	**RUBY**	Addgene 160 908 (He, Yubing, 2020)	p35S-*RUBY* (*CYP76AD1*, *DODA*, and *Glucosyltransferase*)
	**GFP**	pAGM4723 (Marillonnet S, 2011)	p35S-*Clover* (*GFP*)
	**GUS**	pAGM4723 (Marillonnet S, 2011)	p35S-*GUS*
	**Cas9-Ant1**	pAGM4723 (Marillonnet S, 2011)	p35S-*hCas9*, pAtU6-sgRNA (*SlANT1* target)
	**Cas9-Psy1**	pAGM4723 (Marillonnet S, 2011)	p35S-*hCas9*, pAtU6-sgRNA (*SlPsy1* target)
**Replicon**	**GUS**	pLSL.R.Ly (Vu, Tien Van, 2020)	p35S-*GUS*
	**GFP**	pLSL.R.Ly (Vu, Tien Van, 2020)	p35S-*Clover* (*GFP*)
	**Cas9-THCAS**	pLSL.R.Ly (Vu, Tien Van, 2020)	p35S-*hCas9*, pAtU6-sgRNAs (Cs*THCAS* targets)

## Results

### Validation of Tv system in *Agrobacterium* cell

**Figure 1:**
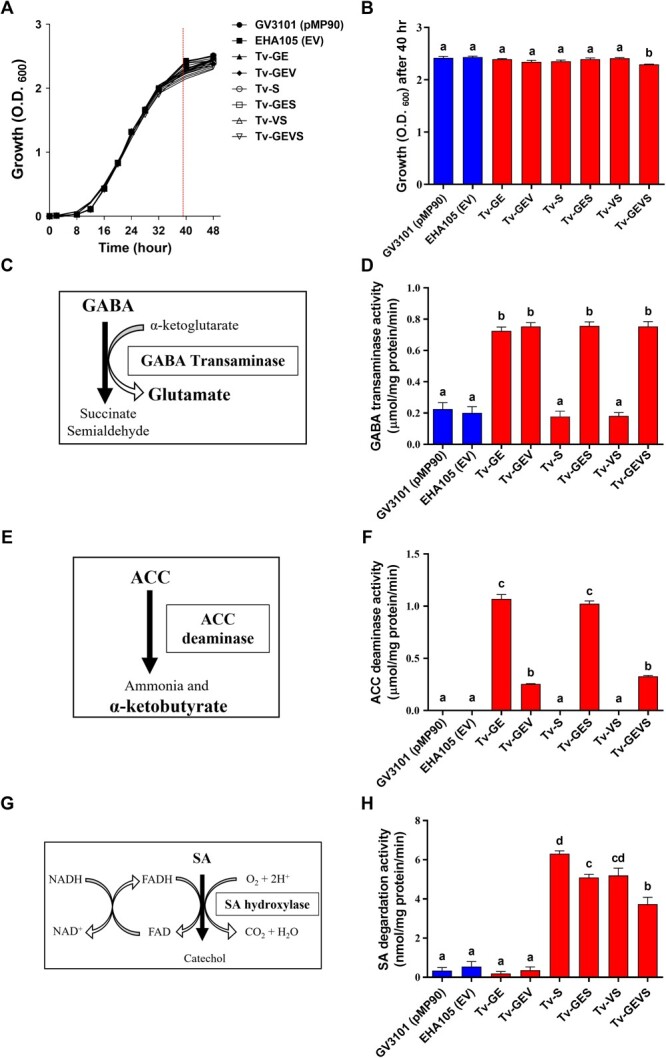
Validation of ACC deaminase, GABA transaminase, and salicylate hydroxylase in *A. tumefaciens.*  **(A)** Effect of ternary vectors on *Agrobacterium* growth. **(B)** Comparison of *Agrobacterium* growth at the end of the exponential stage (40 h). The enzymatic reaction of ACC deaminase **(C)**, GABA transaminase activity **(E)**, and salicylate hydroxylase activity **(G)**. Confirmation of ACC deaminase activity, which measured production of α-ketobutyrate in cell lysates **(D)**, GABA transaminase activity, which measured glutamate production in cell lysates **(F)**, and salicylate hydroxylase activity, which measured decrease in salicylate absorbance in cell lysates **(H).** Data are from at least three independent experiments with biological replicates and are expressed as means ± SD. Different characters indicate a statistically significant difference based on one-way ANOVA and Tukey's multiple range test, with *P* < 0.05.

Ultimately, we developed the super-infective Tv system containing constitutively active VirG and specific enzymes that neutralized signal molecules from plants. For the Tv system, in addition to the disarmed helper plasmid and binary vector, an additional plasmid was introduced into the *Agrobacterium* strain. We validated our system using the hyper-virulent *Agrobacterium* strain EHA105, which is commonly used in plant transformation, and other frequently used strains GV3101 (pMP90) and LBA4404 were also compared as the control strains (Table **S1**)**.** We examined introduced enzyme activities and bacterial growth profiles to confirm whether ternary vectors with introducing foreign genes are compatible in *Agrobacterium*. The virulence gene promoters were used to drive each expression cassette. VirD1 promoter controlled the co-expression cassette of both *GabT* and *AcdS,* and VirG promoter drove the expression of *virG*^N54D^ and *NahG*, respectively (Fig. **S1**). And then, these expression cassettes were combined into pBBR1MCS-5, the broad-host-range plasmid [65] (Fig. **S1A**). Finally, we generated six types of the superior Tv, i.e. *A. tumefaciens* EHA105 including Tv version facilitating GABA (G) and ethylene (E) degradation, a dual-functional version combining GABA-ethylene (GE) degradation with constitutive VirG expression (V), a version specialized in salicylate (S) degradation, two hybrid versions each integrating GABA-ethylene degradation with either salicylate degradation or constitutive VirG expression, and a comprehensive version that amalgamates all the aforementioned components (hereafter referred to as Tv-GE, Tv-GEV, Tv-S, Tv-GES, Tv-VS, and Tv-GEVS, respectively) (Table **1**). In the growth test, most strains showed consistent growth profiles, but Tv-GEVS was significantly inhibited after 40 h, which is the end of the exponential phase (Fig. **1****A, B**). These results indicated that the expression of up to three additional foreign genes does not affect the growth of the strains, while more than a certain number of foreign genes might slightly inhibit the growth of the *Agrobacterium* strain. Colorimetric analyses were performed to examine enzyme activities in *Agrobacterium* cells. The α-ketoglutarate-dependent GabT synthesizes glutamate from GABA [66]. Accordingly, the GabT activity was measured as GABA-dependent glutamate production (Fig. **1****C, D**). AcdS produces ammonia and α-ketobutyrate by reducing 1-aminocyclopropane-1-carboxylic acid (ACC), ethylene precursor, amounts [67]. Therefore, the AcdS activity was measured as the ACC-dependent α-ketobutyrate production (Fig. **1E**). The significantly increased activities of both enzymes were observed in the strains Tv-GE, Tv-GEV, Tv-GES, and Tv-GEVS, carrying both *GabT* and *AcdS* genes, but Tv-GEV and Tv-GEVS showed lower activity than Tv-GE and Tv-GES (Fig. **1****E, F**). The SA-degrading enzyme, salicylate hydroxylase NahG, converts SA to catechol with a cofactor FAD under aerobic conditions [68]. For measurement of salicylate hydroxylase activity, the remaining SA after the reaction was quantified with FeCl_3_ by UV-visible spectrometer [69, 70] (Fig. **1G**). The significantly increased activity of salicylate hydroxylase was detected in the strains Tv-S, Tv-GES, Tv-VS, and Tv-GEVS carrying the *NahG* gene compared with the controls. The strain Tv-S carrying a single gene showed ~20, 20, and 30% higher activities than in Tv-GES, Tv-VS, and Tv-GEVS, respectively (Fig. **1H**). These results show that the enzyme activity was validated in our Tv system.

### Tv system dramatically enhances the *Agrobacterium*-mediated transient transformation

**Figure 2:**
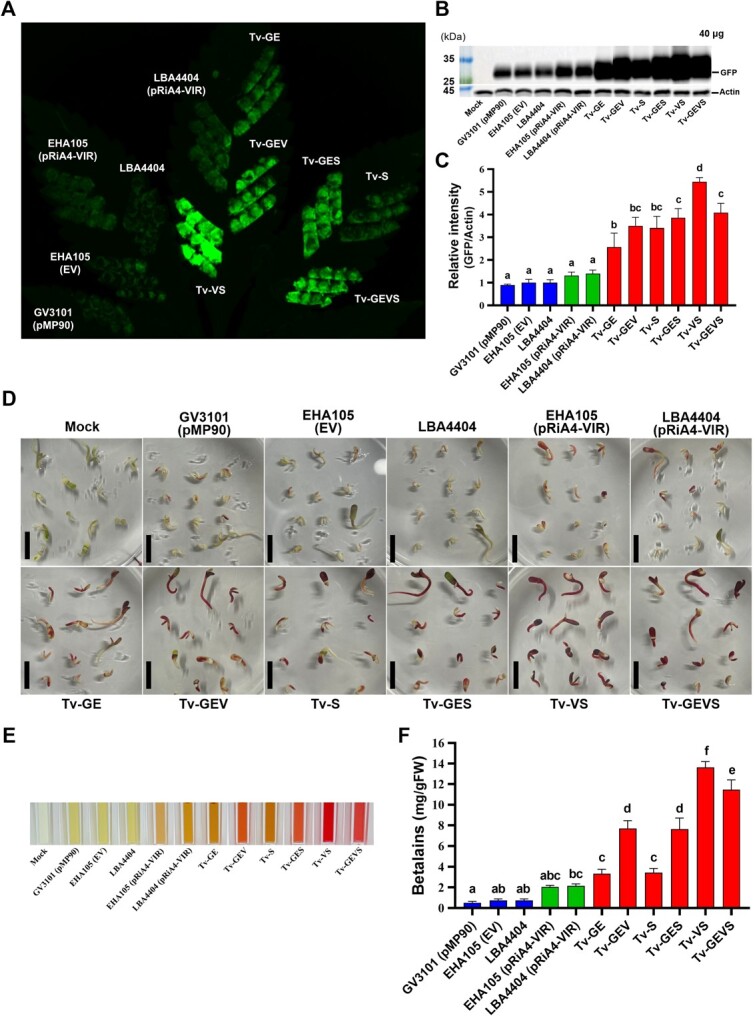
Transient transformation assay in *C. sativa.*  **(A)** Visualization of Clover GFP expression in *C. sativa* leaf using Azure 600 Imaging system*.*  **(B)** Representative western blot analysis with anti-GFP antibodies was performed in *C. sativa* leaf. Actin served as loading control. **(C)** Quantification of GFP protein levels in *C. sativa* using ImageJ. Data are from at least three independent experiments with biological replicates and are expressed as means ± SD. These plants were incubated for 4 days after agro-infiltration using *Agrobacterium* strains carrying *GFP* construct. The visible RUBY expression **(D)** and extraction of betalain pigments **(E)** from RUBY-transformed *Cannabis*-immature embryos. **(F)** Betalain contents were measured spectrophotometrically in extracts of *Cannabis* embryos. Data are from at least three independent experiments with biological replicates and are expressed as means ± SD. These embryos were incubated for 3 days after co-cultivation using *Agrobacterium* strains carrying *RUBY* construct. Different characters indicate a statistically significant difference based on one-way ANOVA and Tukey's multiple range test, with *P* < 0.05.

To confirm whether the Tv systems improved the expression of the protein of interest, *Agrobacterium*-mediated transient gene expression was detected in *N. benthamiana* and *C. sativa*. A binary vector harboring an expression cassette of *Clover*, an improved *GFP* variant, was introduced into all the strains. Subsequently, the fluorescence signal and GFP protein level were compared in the leaf of *C. sativa* (Fig. **2****A, B**). The leaf spots infiltrated by all of the Tv versions had enhanced GFP signals compared to that of strain GV3101 (pMP90), EHA105 (EV), LBA4404, and even strains containing pRiA4-VIR, which include Vir regulon [52, 71] (Fig. **2A** and **S2**). Then, expressed GFP protein in *C. sativa* and *N. benthamiana* was detected by immunoblot analysis with anti-GFP antibodies. Similarly, the expression of GFP protein in all Tv versions was higher than that of control strains (Fig. **2****B, C** and **S3**). Notably, the best expression was seen in Tv-VS, which expresses *virG*^N54D^ and *NahG*. Additionally, we evaluated the transient transformation with Tv system in the *Cannabis*-immature embryos. Reporter RUBY, carrying three genes to convert tyrosine to betalain, was used as the visible selection marker [72]. After transformation, the betalain pigment accumulation in embryos and their extracts was enough to identify the transformation rate. All the Tv systems showed enhanced RUBY expression compared to GV3101 (pMP90), EHA105 (EV), LBA4404, and strains containing pRiA4-VIR, with stronger pigmentation in Tv-GEV, Tv-GES, Tv-VS, and Tv-GEVS (Fig. **2****D-F**). Importantly, we noted that Tv versions harboring a novel component, salicylate hydroxylase *NahG,* when combined with *virG*^N54D^, showed the best performance in transient assays.

### Tv system is compatible with a T-DNA system carrying a viral replicon

**Figure 3:**
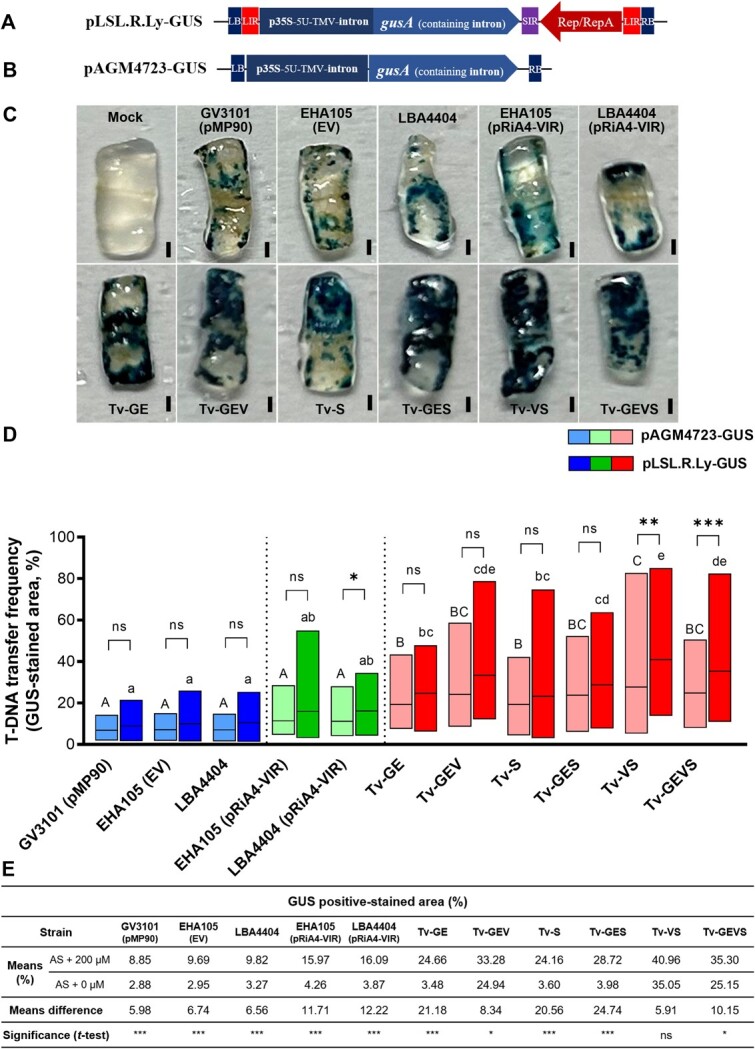
Evaluation of T-DNA transfer frequency with geminivirus-based replicon system in Tv system. GUS expression cassettes in T-DNA region of the geminivirus-based replicon **(A)** and T-DNA based conventional binary vector **(B). (C)** GUS-stained explants of tomato cotyledons after *Agrobacterium*-mediated transient transformation by the geminivirus-based replicon. **(D)** The percentage of GUS-stained area was evaluated in tomato cotyledons by conventional T-DNA based binary vector and geminivirus-based replicon. Values are means ± SD (*n* = 36). Different characters indicate a statistically significant difference based on one-way ANOVA and Tukey's multiple range test, with *P* < 0.05. Asterisks indicate a statistically significant difference based on one-way ANOVA and Tukey's multiple range test, with ^*^P < 0.05, ^**^P < 0.01, and ^***^P < 0.001. **(E)** Comparison of the GUS-stained percentage of each strain in the presence or absence of AS. Values are means ± SD (*n* = 36). Asterisks indicate a statistically significant difference based on unpaired *t*-test, with ^*^P < 0.05 and ^***^P < 0.001. *Agrobacterium* strains contained *GUS*-construct. Scale bars in images represent 1 mm.

The bean yellow dwarf virus (BeYDV) replicon has been used to generate large amounts of DNA copies, which engage highly efficient protein expression in plants [73–75]. Since our Tv system significantly enhanced reporter gene expression, the potential of this system with geminivirus-based replicons in tomato was investigated. The pLSL.R.Ly T-DNA plasmid, a *de novo*-engineered geminiviral replicon vector [76] modified for β-glucuronidase (GUS) expression, and a T-DNA based conventional binary vector were both inoculated into tomato cotyledons (Fig. **3A** and **B**). In this geminiviral replicon version, circularized DNA replicon showed the maximum copy number between 3 and 4 days after *Agrobacterium* inoculation [76]. Thus, GUS expression was evaluated at 4 days after each *Agrobacterium* inoculation. After histochemical GUS assays, the GUS-stained area in each of disc was measured by ImageJ (Fig. **3C**). With T-DNA only, all the Tv versions showed that the transformed cells with GUS-stained blue area were significantly increased, compared to GV3101 (pMP90), EHA105 (EV), LBA4404, and strains containing pRiA4-VIR with a significant increase in Tv-GE, Tv-S, Tv-GES = Tv-GEVS = Tv-GEV, and Tv-VS in increasing order (Fig. **3D**). All replicon versions enhanced slightly or significantly compared with T-DNA-only versions, significant enhancements were detected in Tv-VS and Tv-GEVS. In the *Cannabis* leaf infiltration assay, this replicon system also demonstrated higher efficiency (Fig. **S4**). To decipher the relationship between constitutive virG mutant and *vir* regulon-inducer phenolics, we compared the GUS assay in tomato cotyledons to the presence or absence of AS. It is a common practice to apply AS, as a phenolic compound, in the co-cultivation and pre-culture of *Agrobacterium* cells for activation of *Agrobacterium* virulence. In all control Agrobacteria and Tv-GE, Tv-S, and Tv-GES, AS addition dramatically enhanced GUS staining (Fig. **3E**). GUS staining was slightly enhanced in Tv-GEV and Tv-GEVS with a significance, but in Tv-VS without significance. These results indicate that Tv versions containing *virG* constitutive mutant are still moderately responding to phenolics.

### Tv system-mediated DNA delivery enhanced stable transformation with CRISPR/Cas9-mediated genome editing

**Figure 4:**
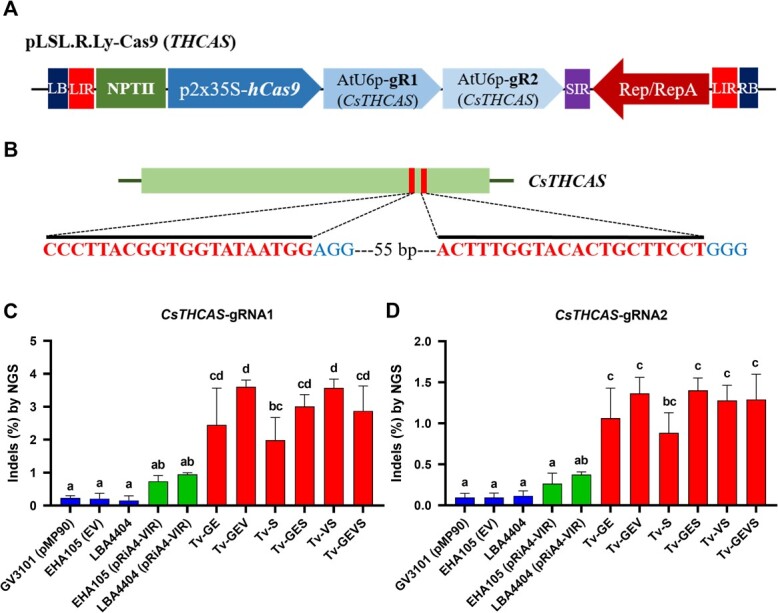
Synergistically increased efficiency of genome editing via Tv system and geminiviral replicon in *C. sativa.*  **(A)** Schematic representation of CRISPR/Cas9 expression with *CsTHCAS*-target gRNAs in T-DNA region of geminivirus-based replicon. Human-optimized SpCas9 (hCas9) and *CsTHCAS*-target gRNAs were driven by 35S promoter with a duplicated enhancer (p2x35S) and AtU6 promoters, respectively. **(B)** Schematic map of the target gene (*CsTHCAS)*. Thin vertical lines represent the target sites within the coding sequence (CDS). The letters below the bar indicate target sequences. Indel frequencies for *CsTHCAS*-gRNA1 **(C)** and -gRNA2 **(D)** in *Cannabis*-immature embryos determined by targeted deep sequencing. Data are from at least three independent experiments with biological replicates and are expressed as means ± SD. Different characters indicate a statistically significant difference based on one-way ANOVA and Tukey's multiple range test, with *P* < 0.05. *Agrobacterium* strains contained *Cas9-THCAS*-construct in replicon.

**Figure 5:**
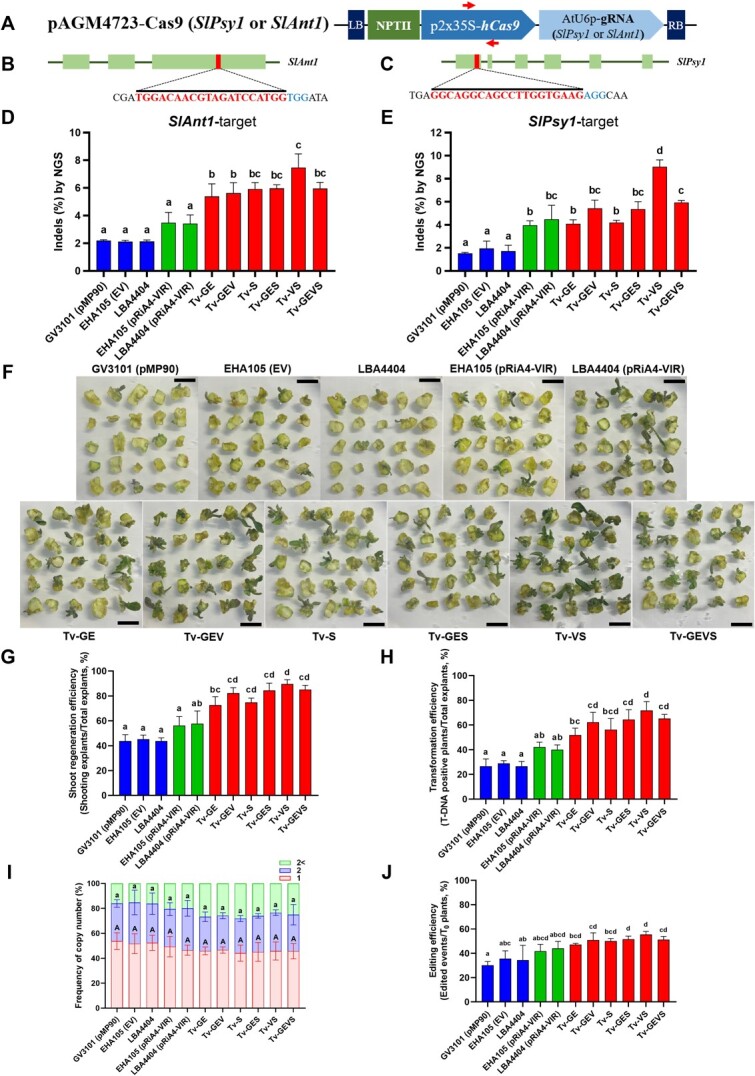
Improvement of genome editing, stable transformation, and regeneration via Tv system. **(A)** Schematic representation of CRISPR/Cas9 expression in T-DNA. 35S with a duplicated enhancer (p2x35S) and AtU6 promoters were used for hCas9 and gRNA, respectively. Red arrows indicate a set of specific primers for T-DNA insertion. Schematic maps of the target genes *SlAnt1*  **(B)** and *SlPsy1*  **(C)**. Thin vertical lines represent the target site within the coding sequence (CDS). The letters below the bar indicate target sequences. Indel frequencies for *SlAnt1*  **(D)** and *SlPsy1*  **(E)** in tomato cotyledons determined by targeted deep sequencing. **(F)** Kanamycin-resistant shoots were regenerated from tomato cotyledons with *Agrobacterium* strains carrying the *Cas9* construct. Scale bars, 1 cm. Effect of Tv system on shoot regeneration **(G)**, transformation efficiency **(H),** and copy number of T-DNA **(I)**. **(J)** Genome editing efficiency was determined by analyzing T-DNA-inserted T_0_ plants for edits in the targeted gene (*SlAnt1*), and the editing was confirmed through Sanger sequencing. Data are from at least three independent experiments with biological replicates and are expressed as means ± SD. Different characters indicate a statistically significant difference based on one-way ANOVA and Tukey's multiple range test, with *P* < 0.05. *Agrobacterium* strains contained *Cas9-*(*Ant1* or *Psy1*)-construct.

CRISPR/Cas9 system has immense potential to plant biotechnology. We tested targeted mutagenesis by CRISPR/Cas9 in tomato and *C. sativa* to assess whether our Tv system can enhance genome editing. In *C. sativa*, tetrahydrocannabinol (THC) is a significant psychoactive constituent, and *THCA synthase* (*THCAS*) serves as a meaningful target for various studies and applications. To efficiently assess *Cannabis* genome editing, we employed synergistically designed gRNAs targeting *CsTHCAS,* along with a replicon system in *Cannabis*-immature embryos (Fig. **4****A, B**). Notably, the mutagenesis efficiencies observed in all the Tv versions were significantly higher than those in control strains and containing pRiA4-VIR, with the maximum in Tv-GEV and Tv-VS demonstrating a remarkable increase to 3.6% editing efficiency from the 0.2% editing efficiency observed with the control strain EHA105 (Fig. **4****C, D**). This 18-fold increase suggests that the increase of Tv system-mediated CRISPR/Cas9 expression dramatically enhances the editing efficiency in *C. sativa*. Next, we evaluated the abilities of Tv system-mediated genome editing using tomato cotyledons with gRNA targeting the tomato *Phytoene synthase 1* (*SlPsy1*) and *Anthocyanin 1* (*SlAnt1*), respectively (Fig. **5**). Similarly, to the case in *C. sativa*, all the Tv versions showed higher editing efficiency in the *SlAnt1*-target construct, with maximum efficiency in Tv-VS (Fig. **5D**). In both the *SlAnt1* and *SlPsy1*-target constructs, Tv-VS exhibited remarkably improved editing efficiency compared with other Tv systems (Fig. **5****D, E**).

Given that *SlANT1* is involved in anthocyanin biosynthesis regulation, we decided to observe the regeneration process and subsequently validated the stable integration of T-DNA in tomato genome for stable transformation [77–79]. To comprehensively identify the ability of the Tv system to transform tomato, we scored the number of the callus-forming explants, regenerated shoots, rooted shoots, and regenerated intact plants (Table **S3**). The explants were able to develop compact calli and initiated shoots 4 weeks after inoculation. Shoot regeneration efficiencies with all the Tv versions were significantly increased compared to control strains (Fig. **5****F, G**). In contrast, the rooting rate in regenerated shoots was observed similarly in all the strains. Finally, we successfully obtained regenerated, intact plants from different explants, and polymerase chain reaction (PCR) analysis confirmed the integration of T-DNA into the plant genomes. Notably, among the strains tested, Tv-VS emerged as the most effective, demonstrating a remarkable 248% increase in T-DNA transformation efficiency when compared to control strains (Fig. **5H**). Additionally, our observations revealed that across all strains, there was a consistent presence of both single and double copies of T-DNA integration (Fig. **5I**). Thus, the Tv system significantly increase stable transformation rates, but not transgene copy number. When examining the genome editing in T-DNA-inserted regenerated plants, we found that the increased stable transformation efficiency provided by the Tv system resulted in a higher number of genome-edited plants (Fig. **5J** and Table **S4**). Furthermore, we evaluated the stable genetic transformation with the Tv system with RUBY reporter in the *A. thaliana*. After floral dipping transformation, T_1_ seeds from each strain-dipped plant were harvested. Transgenic seeds could be observed easily, providing dark red or pink colors (Fig. **S5**). Transformation efficiencies of all the Tv systems were increased compared to control strains, and that of Tv-VS was significantly increased (Fig. **S5**). These observations suggest that our Tv system is also a good toolbox for *in planta* transformation.

## Discussion

As part of its infection strategy, *Agrobacteria,* plant-pathogenic bacteria, can deliver DNA to plant cells. The progress of research in *A. tumefaciens* has facilitated the use of tumor-inducing T-DNA, enabling the cloning of desired genes for plant transformation (Fig. **S6**). Nevertheless, in *Agrobacterium–*plant interaction, the accumulation of SA, IAA, and ethylene inhibits the expression of virulence genes via interrupting VirA/VirG two-component system. Besides, GABA and SA suppress the *Agrobacterium* quorum-sensing signal via the activation of the *attKLM* operon (Fig. **S7**). Therefore, the putative solutions to overcome these negative influences and efforts to improve plant infection efficiency should be required.

**Figure 6:**
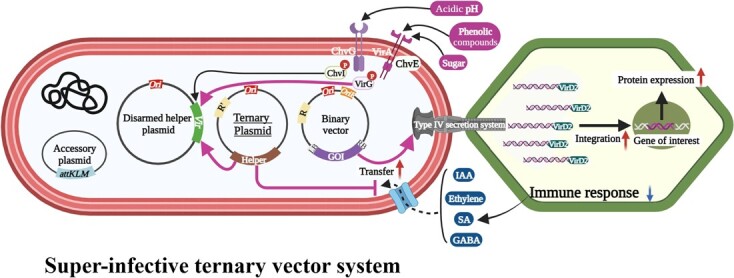
Schematic representation of super-infective ternary vector system. *Agrobacterium* perceives plant signals, such as neutral sugars, phenolic compounds, and an acidic pH, leading to the induction of *Agrobacterium* virulence gene expression. In contrast, *Vir* gene expression is inhibited by negative factors, such as ethylene, IAA, SA, and GABA. VIR proteins play a crucial role in the T-DNA transfer processes facilitated by the type IV secretion system. The successful integration leads to the expression of the T-DNA-encoded genes. In the super-infective ternary vector system**,** a third plasmid is introduced, carrying the host defense-avoidance mechanisms, and promoting *Vir* gene induction, thus dramatically increasing the transformation efficiency.

The development of the super-infective Tv system presented in this study represents a major breakthrough in overcoming the limitations of *Agrobacterium*-mediated transformation, particularly for recalcitrant crops. By strategically combining the overexpression of crucial virulence genes (*virG*^N54D^) with the degradation of plant defense signals (GABA, Ethylene, and SA) through specific enzymes (GabT, AcdS, and NahG), this system achieves remarkable enhancements in transient and stable transformation across diverse plant species and techniques (Fig. **6**). Specifically, in plants like *C. sativa*, where not just transformation but also genome editing has been notably challenging, the observed increase in genome editing efficiency by >18-fold with the Tv system opens up new avenues for R&D possibilities in Cannabis NGTs. This advancement underscores the Tv system's potential in facilitating transformation and significantly boosting gene editing efficiency in species traditionally considered intractable, thereby broadening the scope for NGT applications.

*Agrobacterium* signaling networks are highly complex, interacting with a variety of plant metabolites that can influence the bacterium's response both individually and in combination [23, 60]. To navigate this complexity, we developed several combinations of Tv systems. Our results show that these systems allow for diverse approaches to enhance plant transformation efficiency. The observed dominance of Tv-VS in most assays highlights the synergistic effect of virG^N54D^ and NahG. While virG^N54D^ bypasses the VirA-mediated activation step, directly promoting Vir gene expression, NahG effectively dismantles the SA-triggered quorum-sensing system that suppresses virulence. This combined approach seems to disarm the plant's innate defense system, creating a more favorable environment for T-DNA transformation.

The reason why Tv-GEVS, which includes all components, did not show the best performance could be due to the broader impact of SA on *Agrobacterium* virulence compared to GABA or ethylene. Indeed, Tv-S, which carries a single gene, showed similar efficiency to Tv-GE, which carries both GabT and AcdS genes. Another potential reason for Tv-VS outperforming Tv-GEVS could be the metabolic burden imposed by the additional gene constructs in Tv-GEVS, leading to reduced overall fitness and efficiency. Indeed, bacterium growth was also inhibited in Tv-GEVS (Fig. **2A** and **B**). *GabT* and *AcdS* are expressed polycistronically, but when virG is constitutively expressed, the metabolic burden reduces the activity of AcdS, which is located downstream (Fig. **2F**). This suggests that an optimal balance between the number and type of introduced genes is crucial for maximizing transformation efficiency. Future studies could explore tailored Tv systems that optimize gene combinations for specific plant species and conditions.

The Tv system's modular design, allowing for different combinations of components, is a key strength. This versatility opens up exciting possibilities for tailoring the system to specific plant hosts and their unique defense mechanisms. For instance, plants with high basal SA levels or those that produce a surge of SA during infection might benefit more from Tv versions containing NahG, like the highly efficient Tv-VS. Thus, we cannot exclude the possibility that other versions may have higher transformation efficiencies in different plant hosts, organs, and infection conditions. For example, other combination versions might have a better effect in plants with high GABA content than Tv-VS. This adaptability can be immensely valuable for tackling previously recalcitrant crops, expanding the horizons of plant biotechnology.

Recently, additional desired traits for *Agrobacterium* have been identified, including the heterologous expression of *Pseudomonas syringae* effector proteins, which have been found to enhance transformation efficiency through the use of the type III secretion system [80]. This advancement underscores the need for our Tv system to adapt and utilize these novel characteristics. Furthermore, using a combination approach, the new version of Tv can be easily updated as required.

In conclusion, the super-infective Tv system represents a significant advancement in *Agrobacterium*-mediated transformation. Its adaptability, efficiency, and capability to address challenging crops position it as a pivotal innovation in plant biotechnology. Further investigation into its operational mechanisms and specific applications, along with the exploration of its extensive potential, is poised to herald a new era of crop breeding.

## Materials and methods

### Bacterial strains, media, and growth conditions

*Escherichia coli* DH5α chemically competent cells were used as cloning host. *Escherichia coli* strains were grown at 37°C in the Luria Bertani (LB) medium (5 g/l yeast extract, 10 g/l tryptone, 10 g/l NaCl, and 15 g/l agar) with the following final concentrations of antibiotics: 10 μg/ml gentamicin or 50 μg/ml kanamycin. *Agrobacterium tumefaciens* used in this study are detailed in Table **S1**. *Agrobacterium* strains were grown at 28°C on a liquid medium for yeast extract peptone (YEP) medium (10 g/l yeast extract, 10 g/l bacto peptone, and 5 g/l NaCl) and solid medium for LB medium with the following antibiotic concentrations: 50 μg/l rifampicin, 10 μg/l gentamicin, 50 μg/l kanamycin, and 50 μg/l spectinomycin.

### Ternary vector constructions

The broad-host-range plasmid, pBBRMCS-5B, was used to generate all the ternary vector versions (Fig. **S1**). The sequences of *GabT* gene, *AcdS* gene, and *NahG* gene were found to be identical to accession numbers 948 067, AY823987, and M60055.1 in NCBI database (http://www.ncbi.nlm.nih.gov), respectively. The co-expression cassette of *GabT* and *AcdS* was produced by a gene synthesis service (GenScript Inc.), and the expression cassette of *NahG* was produced by a gene synthesis service (Genewiz Inc.). *virG*^N54D^ was amplified from Addgene vector 123 187. For Tv-GE (pBBR1-*GabT/AcdS*), the co-expression cassette of *GabT* and *AcdS* was amplified using the primers GabT-AcdS-F1 and GabT-AcdS-R1. For Tv-GEV (pBBR1-*GabT/AcdS/virG*^N54D^), the co-expression cassette of *GabT* and *AcdS* and the expression cassette of *virG*^N54D^ were amplified using the primer sets GabT-AcdS-F1/GabT-AcdS-R2 and virG-F2/virG-R1, respectively. For Tv-S (pBBR1-*NahG*), the expression cassette of *NahG* was amplified using the primers virG-F1 and virG-R1. For Tv-GES (pBBR1-*GabT/AcdS/NahG*), the co-expression cassette of *GabT* and *AcdS* and the expression cassette of *NahG* were amplified using the primer sets GabT-AcdS-F1/GabT-AcdS-R2 and virG-F2/virG-R1, respectively. For Tv-VS (pBBR1-*virG*^N54D^*/NahG*), the expression cassette of *virG*^N54D^ and the expression cassette of *NahG* were amplified using the primer sets virG-F1/NahG/virG-R1 and NahG/virG-F1/virG-R1, respectively. For Tv-GEVS (pBBR1- *GabT/AcdS/virG*^N54D^*/NahG*), the co-expression cassette of *GabT* and *AcdS*, the expression cassette of *virG*^N54D^, and the expression cassette of *NahG* were amplified using the primer sets GabT-AcdS-F1/GabT-AcdS-R2, virG-F2/NahG/virG-R1, and NahG/virG-F1/virG-R1, respectively. Used primers are listed in Table **S2**. All the amplicons were cloned into pBBRMCS-5B via Golden gate assembly to generate super-infective ternary vectors. These correct clones were verified by Sanger sequencing (Solgent Co. Ltd). All the Tv versions and pBBR1-MCS-5B were introduced into *A. tumefaciens* strain EHA105 via electroporation.

### Binary vector constructions

Binary vectors used in this study are detailed in Table **1**. For GFP, GUS, and Cas9 constructs, the overall design principle and cloning procedures followed the Modular cloning (MoClo) system and Golden gate assembly [81, 82]. The gusA gene encodes β-glucuronidase for the GUS construct, which was amplified from Addgene vector 50 332. The Clover GFP gene was amplified from Addgene vector 40 259 for the GFP construct. The 20-nt guide RNA sequence (gRNA) was designed by CRISPR RGEN Tools online software, which was driven by an AtU6 promoter and terminated by 7-T chain sequences. The oligonucleotide sequences for the gRNA construct were commercially synthesized (Table **S2**). The expression cassettes were combined with binary vectors through golden gate cloning.

### ACC deaminase (AcdS) activity assay

*Agrobacterium* strains were grown in the YEP medium until the mid-exponential phase, and then the cells were gathered and resuspended with a DF medium containing 3 mM ACC as the sole nitrogen source [83]. After 1 day of incubation, the cells were washed with 1.5 ml Tris–HCl pH 7.0 buffer and transferred to new tubes. They were resuspended in 600 μl of 0.1 M Tris–HCl pH 8.5 and lysed by adding 30 μl toluene. At this time, toluenized cells were used for the Bradford assay [84] and ACC deaminase activity assay [67], which was modified slightly. For the reaction, 200 μl of toluenized cells were placed in fresh e-tubes and incubated with 20 μl of 0.5 M ACC for 30 min at 30°C. The reaction stopped after adding 1 ml of 0.56 M HCl. The mixture was centrifuged for 5 min at 13 000 rpm at RT. 100 μl of the supernatant was vortexed with 800 μl of 0.56 M HCl, and then 300 μl of 0.2% 2,4-dinitrophenylhydrazine were added. After 30 min of incubation, 2 ml of 2 N NaOH were added. The absorbance of the mixture was measured at 540 mm.

### GABA transaminase (GabT) activity assay

*Agrobacterium* strains were grown in the YEP medium until the mid-exponential phase, and then the cells were gathered by centrifugation. They were lysed by BugBuster master mix (Novagen Inc.) and protease inhibitor cocktail (Roche Inc.). Protein concentration in the lysate was determined by BCA protein assay kit (Thermo Scientific). The GabT activity was measured as glutamate production [66]. For the enzymatic reaction, the lysate sample (100 μg protein) was mixed with prepared assay buffer [0.1 M bicine–NaOH (pH 8.6), 1 mM pyridoxal phosphate (Sigma-Aldrich Co.), 10 mM 2-ketoglutarate (Sigma-Aldrich Co.), 10 mM GABA (Sigma-Aldrich Co.), and a proteinase inhibitor cocktail (Roche Inc.)], and they were incubated at 37°C for 30 min. Following the reaction, glutamate contents were measured using a colorimetric assay using a Glutamate assay kit (Sigma-Aldrich Co.).

### Salicylate hydroxylase (NahG) activity assay

Salicylate hydroxylase activity was assayed according to a modification of the methods of salicylate-dependent hydroxylation reaction [68] and colorimetric assay [70]. *Agrobacterium* strains were grown in the YEP medium until the mid-exponential phase, and then the cells were gathered by centrifugation. They were lysed by BugBuster master mix (Novagen Inc.) and protease inhibitor cocktail (Roche Inc.) and the supernatant was transferred to new e-tubes after centrifugation. Protein concentration in the lysate was determined by a BCA protein assay kit (Thermo Scientific). Fifty micrograms of lysate was used per reaction. The reaction mixture contained 33 mM potassium phosphate buffer (pH 7.0), 200 μM salicylic acid (SA, Sigma-Aldrich Co.), 200 μM β-Nicotinamide adenine dinucleotide hydrate (NADH, Sigma-Aldrich Co.), and 20 μM Flavin adenine dinucleotide disodium salt hydrate (FAD, Sigma-Aldrich Co.). The reaction mixture was incubated at 25°C under aerobic conditions for 30 min. Following the reaction, 100 μl of the reaction mixture, 300 μl of 1% FeCl_3,_ and d 600 μl of dH_2_O were added in a final volume of 1 ml. The NahG activity was measured following remaining SA as a decrease in the absorbance at 560 nm, using UV–Visible spectrophotometry.

### Tomato cotyledon transformation

This study used the tomato cultivar of Hongkwang (a local variety). Tomato seeds were sterilized by immersion in 70% ethanol for 30 s and 3% sodium hypochlorite containing one drop of Triton X-100 for 20 min and washed five times with sterilized distilled water. Following the sterilization, the seeds were germinated on a 1/2MS medium and grown at 25 ± 2°C under 16 L/8 D conditions. Cotyledons from 7-day-old seedlings were used in each experiment.

One cotyledon was excised into two pieces by scalpel for the GUS assay, and 36 explants were subjected to each treatment. The explants were pre-cultured for 24 h. All the *A. tumefaciens* strains were prepared on LB solid medium supplemented with 50 μg/l rifampicin, 10 μg/l gentamicin, and 50 μg/l kanamycin. The bacterial cells were resuspended in ABM-MS liquid medium (pH 5.2) to O.D._600_ = 0.8 with or without 100 μM AS. The pre-cultured explants were inoculated for 20 min and then cultured on ABM-MS solid medium (pH 5.6) at 23°C in the dark for 72 h with or without 100 μM AS. After co-cultivation, the explants were then shifted to a B5-MS medium with timentin for 48 h. They were collected for GUS assay.

For CRISPR/Cas9-mediated genome editing and stable transformation, 60 explants were subjected to each treatment. Co-cultivation was processed as described above. After 3 days of co-cultivation, the explants were placed on a selection medium [containing 0.05 mg/l IAA (Sigma-Aldrich Co.), 0.5 mg/l zeatin, 100 mg/l kanamycin, and 300 mg/l timentin] for 11 days. For NGS analysis, 15 of the explants were collected 14 days after infection. For evaluation of stable transformation, the responded explants were counted and subcultured every 14 days. When the regenerated shoots were elongated sufficiently, they were transferred to a rooting medium [containing 0.1 mg/l NAA, 0.3 mg/l IBA, and 300 mg/l timentin]. The intact plantlets were planted on the soil.

### Agro infiltration

The infiltration was performed in *N. benthamiana* and *C. sativa. Agrobacterium* strains carrying *GFP* construct were grown in LB solid medium. Bacterial cells were harvested and resuspended in 10 mM MES (pH 5.5), 10 mM MgCl2, and 200 μM AS to O.D._600_ = 0.6. They were incubated for 3 h at RT. For *N. benthamiana*, the abaxial surface of tobacco leaves was infiltrated using a 1-ml syringe. *C. sativa* was more compartmented by the veins (Fig. **2a**). Thus, the small region between the veins in the abaxial surface of leaves was infiltrated using a 1-ml syringe 10 times. After 3 days after infiltration, GFP expression was detected by the Azure 600 Imaging system (Azure Biosystems).

### Confocal microscopy

Cannabis leaf sections were imaged using an Olympus FV1000MPE confocal microscope post-*Agrobacterium* infiltration. GFP fluorescence was excited at 488 nm and emissions were collected between 505 and 550 nm. ImageJ software was employed for image processing and analysis.

### Western blotting

The leaves of *N. benthamiana* and *C. sativa* were harvested 4 days after infiltration for protein detection. One hundred milligrams of infiltrated leaves were ground to the fine powder with liquid nitrogen using a mortar and pestle and then mixed with 200 μl of lysis buffer [containing 100 mM Tris–HCl (pH 7.5), 150 mM NaCl, 1 mM EDTA, 0.5% Nonidet P-40 (NP40, Amresco), 2 mM NaF (Sigma-Aldrich Co.), 1.5 mM Na3Vo4 (Sigma-Aldrich Co.), 3 mM 1,4-Dithiothreitol (DTT, Sigma-Aldrich Co.), 1 mM phenylmethanesulfonyl fluoride (PMSF, Sigma-Aldrich Co.), 50 μM MG-132 (Sigma-Aldrich Co.), 1X protease inhibitor cocktail (PIC, Roche Inc.)]. They were incubated on ice for 10 min and centrifuged at 13 000 rpm at 4°C for 15 min. The supernatant was transferred to a fresh tube. The total protein concentration was determined by the Bradford assay [84]. The crude extracts (50 μg of total protein) were mixed with 6× loading dye [containing 375 mM Tris–HCl (pH 6.8), 50% glycerol, 9% sodium dodecyl sulfate (SDS), 0.03% bromophenol blue, 2% β-mercaptoethanol]. They were incubated at 90°C for 5 min and then kept on ice. The samples were applied onto an SDS-PAGE gel. The protein was transferred to a polyvinylidene difluoride membrane (Amersham Hybond P PVDF, GE Healthcare). The membrane was stained with ponceau S staining solution, followed by a brief rinse in distilled water. After scanning, the membrane was probed with a rabbit polyclonal anti-GFP antibody (1:10 000, Abcam-ab6556), followed by the secondary antibody-anti-rabbit IgG (H + L), horseradish peroxidase (HRP) conjugate (1:20 000, Promega-W4011). Protein was detected by the ECL detection reagent (Bio-Rad) and Azure 600 Imaging system (Azure Biosystems). Band intensities were analyzed using ImageJ.

### GUS histochemical analysis

The histochemical analysis of *gusA* gene expression was performed on the transformed tomato cotyledon explants. The explants were collected directly in a fixative solution containing 4% paraformaldehyde for 30 min and washed twice with 100 mM phosphate buffer (pH 7.0) for 1 h. They were incubated overnight at 37°C in a GUS solution [containing 100 mM phosphate buffer (pH 7.0), 0.4 mM potassium ferricyanide, 0.4 mM potassium ferrocyanide, 0.1% Triton X-100, and 1 mM 5-Bromo-4-chloro-3-indolyl-beta-D-glucuronic acid (X-gluc, Thermo Scientific)]. After GUS staining, the stained explants were cleared with 70% ethanol to remove the chlorophyll. GUS expression of explants was visually observed and photographed. The GUS-stained area was calculated by Image J (National Institutes of Health).

### Immature embryo transformation

*C. sativa* cultivar Afghani kush, which was obtained from Sensible Gifts LTD. (UK), was used in this study. For the acquisition of immature embryos, *Cannabis* seeds were initially grown under long-day conditions (16 L/8D) for ~1 month. Subsequently, they were transferred to short-day condition (12 L/12D), which initiated the flowering process. Immature seeds were collected from female plants ~21 days after pollination. Their sterilization followed the tomato seed sterilization procedure. Then, immature embryos were rescued and subsequently used for *Agrobacterium*-mediated transformation. The preparation of *Agrobacterium* was processed as described above. The rescued immature embryos were inoculated for 40 min under vacuum treatment and then cultured on ABM-MS solid medium (pH 5.6) at 23°C in the dark for 72 h with 100 μM AS. After co-cultivation, the explants were then shifted to a 1/2 MS-medium with timentin. For NGS analysis, the explants were collected 7 days after co-cultivation.

### Betalain extraction

The RUBY-transformed immature embryos of *C. sativa* were pooled. The 10 similar-sized embryos were homogenized to a fine powder in liquid nitrogen. Then, betalains were extracted in 1 ml of 80% (v/v) methanol containing 50 mM sodium ascorbate as described in Schliemann et al. [85]. Betalain content of the extracts was determined spectrophotometrically [86].

### Genomic DNA extraction

The method was modified using the SDS/urea-based protocol from Liu et al [87]. The extraction buffer was modified with a minor modification, composed of 10 mM Tris–HCl (pH 7.5), 50 mM EDTA, 0.35 M NaCl, 2% SDS, and 7 M urea. Following the extraction steps, gDNA was eluted in TE-RNase. The DNA concentration was measured using Nanodrop (ND-1000, Thermo Scientific).

### Next generation sequencing (NGS) and Sanger sequencing analysis

NGS analysis followed the procedure by Oh, Lee et al [88]. Following the DNA extraction, target loci of the genomic region harboring the 20-nt gRNA were amplified by PCR with specific primers (Table **S2**). After three steps of PCR (first nested PCR, second PCR, and third PCR with index primers), targeted deep sequencing of PCR amplicons was performed in the KAIST BIOCORE center (http://biocore.kaist.ac.kr/) with Illumina Miniseq (Illumina, USA). The frequencies of Indel mutation were determined using the Cas-Analyzer tool, which is available at CRISPR RGEN Tools [89].

After DNA extraction, PCR is performed using specific primers (Table **S2**) for the first nested PCR that includes a 20-nt target region. The PCR product is then purified using the Exo-CIP Rapid PCR Cleanup kit (NEB). Sanger sequencing is performed by CosmoGenetech (Korea, Daejon).

### Molecular analysis

Total genomic DNA was prepared from fresh leaves of regenerated intact plantlets, as described above. PCR analysis was performed to detect the presence of the *Cas9* gene with a predicted fragment size of 429 bp. Primer sets of *Cas9* for T-DNA integration and *GAPDH* for internal control are listed in Table **S2**. Binary vector pAGM4723-Cas9 was used as a positive control. PCR was carried out in 20 μl reaction volume [containing 100 ng gDNA, 0.2 mM dNTPs, 0.2 μM of each primer, 1X *Taq* Reaction buffer (Solgent Co. Ltd), 5 U DiaStar *Taq* DNA polymerase (Solgent Co. Ltd)]. The PCR reaction mixtures were subjected to the following amplification program with 95°C for 3 min, followed by 35 cycles of 95°C for 20 s, 60°C for 30 s, 72°C for 30 s, and finally 72°C for 5 min. The amplified products were separated by electrophoresis on 1.2% agarose gels stained with SYBR-safe DNA gel stain (Invitrogen) and then confirmed in UV light.

### Quantitative PCR for T-DNA copy number estimation

For quantitative PCR (qPCR) analysis, an Eco™ Real-Time PCR system was utilized. The reaction mix, totaling 10 μl, comprised 5 μl of KAPA SYBR FAST qPCR Master Mix (1x), 0.2 μM of each primer, and 1 μl of genomic DNA, estimated at ~1000 copies/μl based on a tomato genome size of ~950 Mb [90], translating to 10.41 ng/μl. Primers targeting the T-DNA *Cas9* and *LAT52*, which serve as a single-copy reference gene [91], are detailed in Supplemental Table S2. The qPCR protocol entailed a single cycle of DNA denaturation at 95°C for 10 min, followed by 40 cycles of 95°C for 15 s and 60°C for 1 min, concluding with a melting curve analysis from 60°C to 95°C. Both the target and reference gene assays were conducted in triplicate, and the T-DNA copy number was determined using the ΔΔCt method [92].

### *Agrobacterium-*mediated floral dip transformation

The *Arabidopsis* floral dipping transformation was performed as previously described [93]. *Arabidopsis* Columbia (Col-0) was transformed with each *Agrobacterium* strain that harbored binary vector carrying *RUBY* construct. Transgenic seeds were counted by observing the seed's dark red or pink color (Table S5) [72].

### Statistical analysis

The average values were recorded in at least three replicates. Statistical analyses were performed using Prism 5 software (GraphPad). In Figs. **1**, **2**, **3D**, **4**, **5**, **S3**, and **S5**, multiple comparisons were performed by one-way analysis of variance (ANOVA) and Tukey's multiple range test, with *P* < 0.05. In Fig. **3E**, comparisons of the means of each strain in the presence or absence of AS were performed by unpaired *t*-test with ^*^P < 0.05 and ^***^P < 0.001.

## Supplementary Material

Web_Material_uhae187

## Data Availability

All relevant data can be found within the manuscript and its supporting materials.
