# Supplementary material for: Development of super-infective ternary vector systems for enhancing the Agrobacterium-mediated plant transformation and genome editing efficiency
Source: Hortic Res. 2024 Jul 10;11(9):uhae187. doi: 10.1093/hr/uhae187 (PMC11377189; doi:10.1093/hr/uhae187)
Supplement: Web_Material_uhae187 [file web_material_uhae187.zip › Supporting information.docx]

## *Horticulture Research* Supporting Information

Article title: **Development of super-infective ternary vector systems for enhancing the *Agrobacterium*-mediated plant transformation and genome editing efficiency**

Authors: Jin-hee Jeong^1,2*^, Eun-young Jeon^1^, Min Ki Hwang^2^, Young Jong Song^2^ and Jae-Yean Kim^1, 2,3*^

**Author affiliations:**

^1^Nulla Bio Inc., 501 Jinjudaero, Jinju 660-701, Republic of Korea.

^2^Division of Applied Life Science (BK21 Four program), Plant Molecular Biology and Biotechnology Research Center, Gyeongsang National University, Jinju 660-701, Republic of Korea.

^3^Division of Life Science, Gyeongsang National University, 501 Jinju-daero, Jinju 52828, Republic of Korea.

The following Supporting Information is available for this article:

**Table S1. List of *Agrobacterium tumefaciens* strains used in this study.**

| *Agrobacterium* Strain | Background | Disarmed helper plasmid | Ti-plasmid derivative | Opine | Antibiotic resistance |
| --- | --- | --- | --- | --- | --- |
| EHA105 | C58 | pTiEHA105 | pTiBo542 | Sucinamopine | Rifampicin |
| GV3101 (pMP90) | C58 | pMP90 | pTiC58 | Nopaline | Rifampicin/Gentamicin |
| LBA4404 | Ach5 | pAL4404 | pTiAch5 | Octopine | Rifampicin/Streptomycin |

**Table S2. List of oligonucleotide primers used for cloning and PCR.**

| **Name** | **Sequence (5’->3’)** | **Description** |
| --- | --- | --- |
| **GabT-AcdS-F1** | cagtcGGTCTCaGGAGTTTATTTCAAATTCGTTATA | Ternary vector cloning |
| **GabT-AcdS-R1** | cagtcGGTCTCaAAGCcaaaaaacccctcaagaccc | Ternary vector cloning |
| **virG-R1** | cagtcGGTCTCaAAGCccgtcttggtggtcagtgtg | Ternary vector cloning |
| **virG-F1** | cagtcGGTCTCaGGAGtagctgtaacctcgaagcgt | Ternary vector cloning |
| **virG-F2** | cagtcGGTCTCatagctgtaacctcgaagcgt | Ternary vector cloning |
| **GabT-AcdS-R2** | cagtcGGTCTCaGCTAcaaaaaacccctcaagaccc | Ternary vector cloning |
| **NahG/virG-F1** | cagtcGGTCTCaTACTtagctgtaacctcgaagcgt | Ternary vector cloning |
| **NahG/virG-R1** | cagtcGGTCTCaAGTAccgtcttggtggtcagtgtg | Ternary vector cloning |
| **Ant1-gR-F1** | CAGTCGGTCTCAATTGTGGACAACGTAGATCCATGGgttttagagctagaaatagcaagttaaaataaggctagtccgttatcaac | gRNA cloning |
| **Psy1-gR-F1** | CAGTCGGTCTCAATTGGGCAGGCAGCCTTGGTGAAGgttttagagctagaaatagcaagttaaaataaggctagtccgttatcaac | gRNA cloning |
| **THCAS-gR1-F1** | CAGTCGGTCTCAATTGCCCTTACGGTGGTATAATGGgttttagagctagaaatagcaagttaaaataaggctagtccgttatcaac | gRNA cloning |
| **THCAS-gR2-F1** | CAGTCGGTCTCAATTGACTTTGGTACACTGCTTCCTgttttagagctagaaatagcaagttaaaataaggctagtccgttatcaac | gRNA cloning |
| **Universal-gR-R1** | CAGTCGGTCTCAAGCGAAAAAAAgcaccgactcggtgccactttttcaagttgataacggactagccttatttt | gRNA cloning |
| **crU6C9-F1** | CAGTCGGTCTCAGGAGtgatcaaaagtcccacatcgatcaggtgatatatagcagcttagttta | gRNA cloning |
| **crU6C9-R1** | CAGTCGGTCTCAcaatcgctatgtcgactctatcattatataaactaagctgctatatatcacc | gRNA cloning |
| **GAPDH-F1** | CCATAACCTAATTTCTCTCTC | Internal control |
| **GAPDH-R1** | GTCATGAGACCCTCAACAAT | Internal control |
| **Cas9-T-F1** | CGCATCTCTCGGAACCTACC | Confirmation of T-DNA integration |
| **Cas9-T-R1** | GGAGATCCAGCGAGGTTAGC | Confirmation of T-DNA integration |
| **Ant1-1^st^-F** | CGGAAGGACAGCTAACGATG | PCR for Mini-seq and Sanger-seq |
| **Ant1-1^st^-R** | CTTGTTGCATGGGTGGTAAA | PCR for Mini-seq and Sanger-seq |
| **Ant1-2^nd^-F** | ACACTCTTTCCCTACACGACGCTCTTCCGATCTCGAGGAGGAACATTGCAAGG | PCR for Mini-seq |
| **Ant1-2^nd^-R** | GTGACTGGAGTTCAGACGTGTGCTCTTCCGATCTCCTCTTCATCTTCTTCAATATCGTC | PCR for Mini-seq |
| **Psy1-1^st^-F** | TGACGTCTCAAATGGGACAA | PCR for Mini-seq |
| **Psy1-1^st^-R** | GCCCAGATAGCCCTTCTTCT | PCR for Mini-seq |
| **Psy1-2^nd^-F** | ACACTCTTTCCCTACACGACGCTCTTCCGATCTCATCTGGAGAACGGACGATG | PCR for Mini-seq |
| **Psy1-2^nd^-R** | GTGACTGGAGTTCAGACGTGTGCTCTTCCGATCTACTCAACAAGCCCAAATTCC | PCR for Mini-seq |
| **THCAS-1^st^-F** | tgatgaacaagagctttcctga | PCR for Mini-seq |
| **THCAS-1^st^-R** | tttgggacacataaggagtcg | PCR for Mini-seq |
| **THCAS-2^nd^-F** | ACACTCTTTCCCTACACGACGCTCTTCCGATCTaggagctgggatgtatgtgt | PCR for Mini-seq |
| **THCAS-2^nd^-R** | GTGACTGGAGTTCAGACGTGTGCTCTTCCGATCTacttcgaacccagtttatatgct | PCR for Mini-seq |
| **LAT52-qPCR-F1** | TTCTTGCCTTTTCATATCCAGACA | qPCR for reference |
| **LAT52-qPCR-R1** | AGACCACGAGAACGATATTTGC | qPCR for reference |
| **hCas9-qPCR-F2** | Catcgactttctcgaggcga | qPCR for T-DNA copy confirmation |
| **hCas9-qPCR-R2** | cattcgtttccggccgtttt | qPCR for T-DNA copy confirmation |

**Table S3. Effect of Tv system on plant regeneration and transformation efficiency in tomato cotyledons.**

| **Exp.** | **Strain** | **No. explants** | **No. callus forming explants** | | **No. explants**  **with shoots** | | **No. rooting**  **shoots** | | **No. plantlets** | | **PCR**  **positive** | |  |
| --- | --- | --- | --- | --- | --- | --- | --- | --- | --- | --- | --- | --- | --- |
| **1st** | Control | 45 | 0 | | - | | - | | - | | - | |  |
|  | GV3101 (pMP90) | 45 | 34 | | 21 | | 18 | | 17 | | 15 | |  |
|  | EHA105 (EV) | 45 | 32 | | 22 | | 17 | | 14 | | 14 | |  |
|  | LBA4404 | 45 | 33 | | 19 | | 16 | | 12 | | 11 | |  |
|  | EHA105 (pRiA4-VIR) | 45 | 38 | | 23 | | 20 | | 20 | | 20 | |  |
|  | LBA4404 (pRiA4-VIR) | 45 | 39 | | 25 | | 22 | | 22 | | 19 | |  |
|  | Tv-GE | 45 | 38 | | 30 | | 29 | | 28 | | 26 | |  |
|  | Tv-GEV | 45 | 39 | | 39 | | 34 | | 34 | | 32 | |  |
|  | Tv-S | 45 | 41 | | 35 | | 31 | | 30 | | 30 | |  |
|  | Tv-GES | 45 | 42 | | 39 | | 38 | | 37 | | 33 | |  |
|  | Tv-VS | 45 | 43 | | 42 | | 39 | | 38 | | 36 | |  |
|  | Tv-GEVS | 45 | 40 | | 40 | | 38 | | 34 | | 31 | |  |
| **2nd** | Control | 45 | 0 | | - | | - | | - | | - | |  |
|  | GV3101 (pMP90) | 45 | 32 | | 17 | | 15 | | 12 | | 10 | |  |
|  | EHA105 (EV) | 45 | 35 | | 19 | | 15 | | 13 | | 13 | |  |
|  | LBA4404 | 45 | 41 | | 19 | | 16 | | 14 | | 11 | |  |
|  | EHA105 (pRiA4-VIR) | 45 | 37 | | 24 | | 21 | | 19 | | 17 | |  |
|  | LBA4404 (pRiA4-VIR) | 45 | 40 | | 22 | | 18 | | 18 | | 16 | |  |
|  | Tv-GE | 45 | 42 | | 32 | | 29 | | 26 | | 21 | |  |
|  | Tv-GEV | 45 | 39 | | 35 | | 33 | | 33 | | 27 | |  |
|  | Tv-S | 45 | 41 | | 32 | | 27 | | 27 | | 23 | |  |
|  | Tv-GES | 45 | 41 | | 40 | | 39 | | 33 | | 28 | |  |
|  | Tv-VS | 45 | 43 | | 40 | | 39 | | 34 | | 31 | |  |
|  | Tv-GEVS | 45 | 41 | | 38 | | 37 | | 33 | | 29 | |  |
| **3rd** | Control | 45 | 0 | | - | | - | | - | | - | |  |
|  | GV3101 (pMP90) | 45 | 28 | | 21 | | 17 | | 17 | | 11 | |  |
|  | EHA105 (EV) | 45 | 29 | | 20 | | 17 | | 16 | | 12 | |  |
|  | LBA4404 | 45 | 30 | | 21 | | 16 | | 15 | | 14 | |  |
|  | EHA105 (pRiA4-VIR) | 45 | 37 | | 29 | | 25 | | 21 | | 20 | |  |
|  | LBA4404 (pRiA4-VIR) | 45 | 38 | | 31 | | 26 | | 20 | | 19 | |  |
|  | Tv-GE | 45 | | 41 | | 36 | | 32 | | 28 | | 23 | |
|  | Tv-GEV | 45 | | 42 | | 37 | | 34 | | 32 | | 25 | |
|  | Tv-S | 45 | | 37 | | 34 | | 31 | | 27 | | 23 | |
|  | Tv-GES | 45 | | 40 | | 35 | | 33 | | 28 | | 26 | |
|  | Tv-VS | 45 | | 42 | | 39 | | 38 | | 32 | | 30 | |
|  | Tv-GEVS | 45 | | 41 | | 37 | | 36 | | 31 | | 28 | |

**Table S4. Comparison of genome editing frequency with *Agrobacterium* strains in T_0_ plants.**

| **Exp.** | **Strain** | **No.**  **T_0_ plants** | **No.**  **no mutation** | **Mutation types** | | | | | | | **Editing efficiency***  **(%)** |
| --- | --- | --- | --- | --- | --- | --- | --- | --- | --- | --- | --- |
|  |  |  |  | **No.**  **chimera** | **No.**  **hetero** | | | **No.**  **biallelic** | | **No.**  **homo** |  |
| **1st** | GV3101 (pMP90) | 15 | 10 | 2 | 2 | | | 1 | |  | 33.33 |
|  | EHA105 (EV) | 14 | 8 | 3 | 1 | | | 1 | | 1 | 42.86 |
|  | LBA4404 | 11 | 6 | 1 | 2 | | | 2 | |  | 45.45 |
|  | EHA105 (pRiA4-VIR) | 20 | 11 | 1 | 4 | | | 3 | | 1 | 45.00 |
|  | LBA4404 (pRiA4-VIR) | 19 | 10 | 3 | 2 | | | 4 | |  | 47.37 |
|  | Tv-GE | 26 | 14 | 3 | 5 | | | 2 | | 2 | 46.15 |
|  | Tv-GEV | 32 | 14 | 6 | 3 | | | 5 | | 4 | 56.25 |
|  | Tv-S | 30 | 15 | 5 | 5 | | | 2 | | 3 | 50.00 |
|  | Tv-GES | 33 | 15 | 6 | 4 | | | 6 | | 2 | 54.55 |
|  | Tv-VS | 36 | 15 | 5 | 3 | | | 7 | | 6 | 58.33 |
|  | Tv-GEVS | 31 | 16 | 4 | 6 | | | 3 | | 2 | 48.39 |
| **2nd** | GV3101 (pMP90) | 10 | 7 | 3 |  | | |  | |  | 30.00 |
|  | EHA105 (EV) | 13 | 9 | 3 |  | | | 1 | |  | 30.77 |
|  | LBA4404 | 11 | 7 | 1 | 2 | | | 1 | |  | 36.36 |
|  | EHA105 (pRiA4-VIR) | 17 | 11 |  | 2 | | | 3 | | 1 | 35.29 |
|  | LBA4404 (pRiA4-VIR) | 16 | 10 | 2 | 2 | | | 1 | | 1 | 37.50 |
|  | Tv-GE | 21 | 11 | 4 | 2 | | | 4 | |  | 47.62 |
|  | Tv-GEV | 27 | 15 | 2 | 6 | | | 3 | | 1 | 44.44 |
|  | Tv-S | 23 | 11 | 2 | 3 | | | 5 | | 2 | 52.17 |
|  | Tv-GES | 28 | 14 | 3 | 5 | | | 3 | | 3 | 50.00 |
|  | Tv-VS | 31 | 14 | 6 | 4 | | | 4 | | 3 | 54.84 |
|  | Tv-GEVS | 29 | 14 | 6 | 5 | | | 2 | | 2 | 51.72 |
| **3rd** | GV3101 (pMP90) | 11 | 8 |  | 2 | | |  | | 1 | 27.27 |
|  | EHA105 (EV) | 12 | 8 |  | 1 | | | 2 | | 1 | 33.33 |
|  | LBA4404 | 14 | 11 | 1 | 1 | | | 1 | |  | 21.43 |
|  | EHA105 (pRiA4-VIR) | 20 | 11 | 2 | 2 | | | 4 | | 1 | 45.00 |
|  | LBA4404 (pRiA4-VIR) | 19 | 10 | 2 | 3 | | | 3 | | 1 | 47.37 |
|  | Tv-GE | 23 | 12 | 2 | 5 | | | 2 | | 2 | 47.83 |
|  | Tv-GEV | 25 | 12 | 6 | 3 | | | 2 | | 2 | 52.00 |
|  | Tv-S | 23 | 12 | 5 | 3 | | | 1 | | 2 | 47.83 |
|  | Tv-GES | 26 | 13 | 3 | 2 | | | 2 | | 6 | 50.00 |
|  | Tv-VS | 30 | 14 | 5 | | 3 | 5 | | 3 | | 53.33 |
|  | Tv-GEVS | 28 | 13 | 4 | | 6 | 2 | | 3 | | 53.57 |

***(The number of all mutation types/the number of T_0_ plants)**

**Table S5. Comparison of transformation frequency with *Agrobacterium* strains carrying *RUBY* construct.**

| **Exp** | **Strain** | **No. harvested seeds** | **No. RUBY-expressing seeds** | **Transformation efficiency (%)** |
| --- | --- | --- | --- | --- |
| **1st** | Control | 2031 | 0 | - |
|  | GV3101 (pMP90) | 2413 | 1 | 0.04 |
|  | EHA105 (EV) | 3579 | 2 | 0.06 |
|  | Tv-GE | 3531 | 20 | 0.57 |
|  | Tv-GEV | 2520 | 20 | 0.79 |
|  | Tv-S | 2398 | 9 | 0.38 |
|  | Tv-GES | 3251 | 21 | 0.65 |
|  | Tv-VS | 1872 | 20 | 1.07 |
|  | Tv-GEVS | 2022 | 8 | 0.40 |
| **2nd** | Control | 2904 | 0 | - |
|  | GV3101 (pMP90) | 2387 | 4 | 0.17 |
|  | EHA105 (EV) | 2036 | 2 | 0.10 |
|  | Tv-GE | 3323 | 10 | 0.30 |
|  | Tv-GEV | 1611 | 12 | 0.74 |
|  | Tv-S | 2260 | 13 | 0.58 |
|  | Tv-GES | 3563 | 22 | 0.62 |
|  | Tv-VS | 2629 | 48 | 1.83 |
|  | Tv-GEVS | 3112 | 23 | 0.74 |
| **3rd** | Control | 3772 | 0 | - |
|  | GV3101 (pMP90) | 2465 | 18 | 0.73 |
|  | EHA105 (EV) | 2871 | 11 | 0.38 |
|  | Tv-GE | 2623 | 19 | 0.72 |
|  | Tv-GEV | 2328 | 18 | 0.77 |
|  | Tv-S | 2851 | 16 | 0.56 |
|  | Tv-GES | 2674 | 18 | 0.67 |
|  | Tv-VS | 2738 | 30 | 1.10 |
|  | Tv-GEVS | 2376 | 18 | 0.76 |


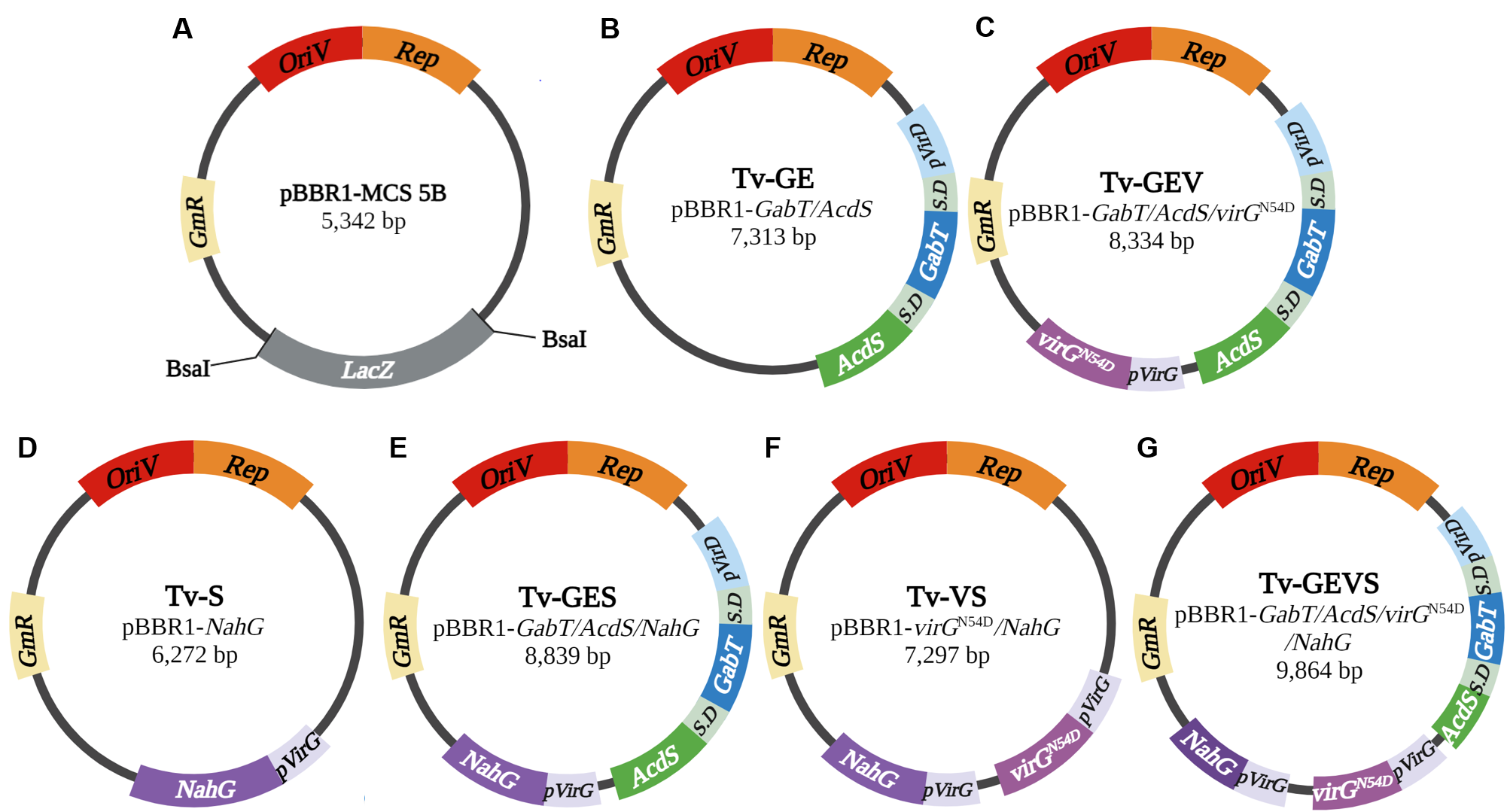


**Figure S1. Vector maps of broad-host-range shuttle pBBR1 and several versions of the Tv system.** Map of plasmid pBBR1-MCS5B backbone **(A)**, Tv-GE; pBBR1-*GabT/AcdS* **(B)**, Tv-GEV; pBBR1-*GabT/AcdS/virG*^N54D^ **(C)**, Tv-S; pBBR1-*NahG* **(D)**, Tv-GES; pBBR1-*GabT/AcdS/NahG* **(E)**, Tv-VS; pBBR1-*virG*^N54D^/*NahG* **(F)**, and Tv-GEVS; pBBR1-*GabT/AcdS/virG*^N54D^/*NahG* **(G)**. *OriV*, replication origin of pBBR1 from *B. bronchiseptica*; *Rep*, protein for replication required by pBBR1 *OriV*; *GmR*, Gentamicin resistance gene.

**Figure S2. Localization of Clover GFP in *Cannabis* leaf plant cells observed through confocal microscopy.** *Cannabis sativa* plants were cultured for 4 days after agro-infiltration using *Agrobacterium* strains carrying *GFP* construct. GFP fluorescence is shown in left panel and bright field is shown in middle panel. The overlay of GFP signals and bright field appears right panel. Scale bars in images represent 50 μm.


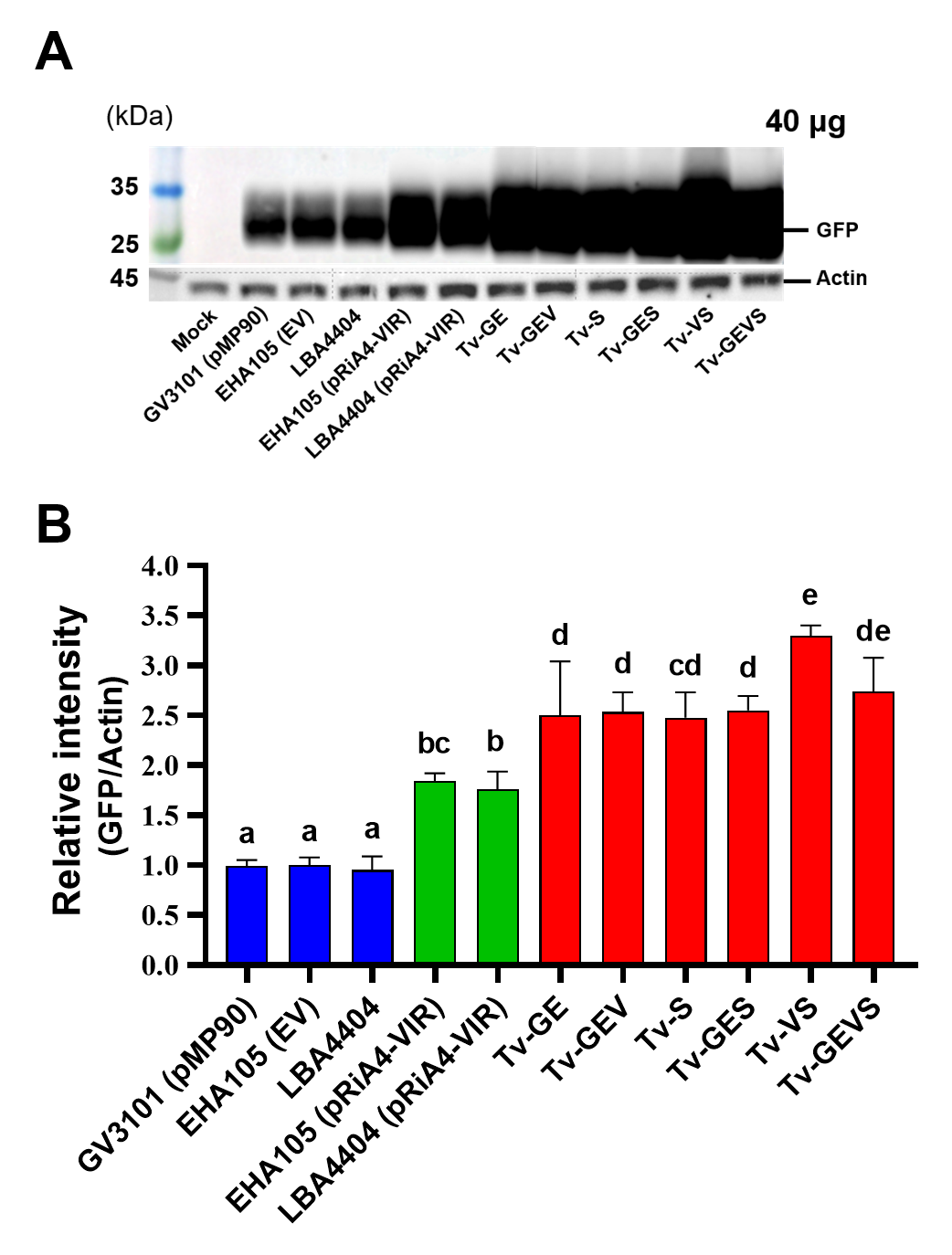


**Figure S3. Transient expression of GFP in *Nicotiana benthamiana****.* **(A)** Representative western blot analysis with anti-GFP antibodies was performed in *N. benthamiana.* **(B)** Quantification of GFP protein levels in *N. benthamiana* using ImageJ. Values are means ± SD (*n* = 3). Actin served as loading control. These plants were incubated for 4 days after agro-infiltration using *Agrobacterium* strains carrying *GFP* construct.


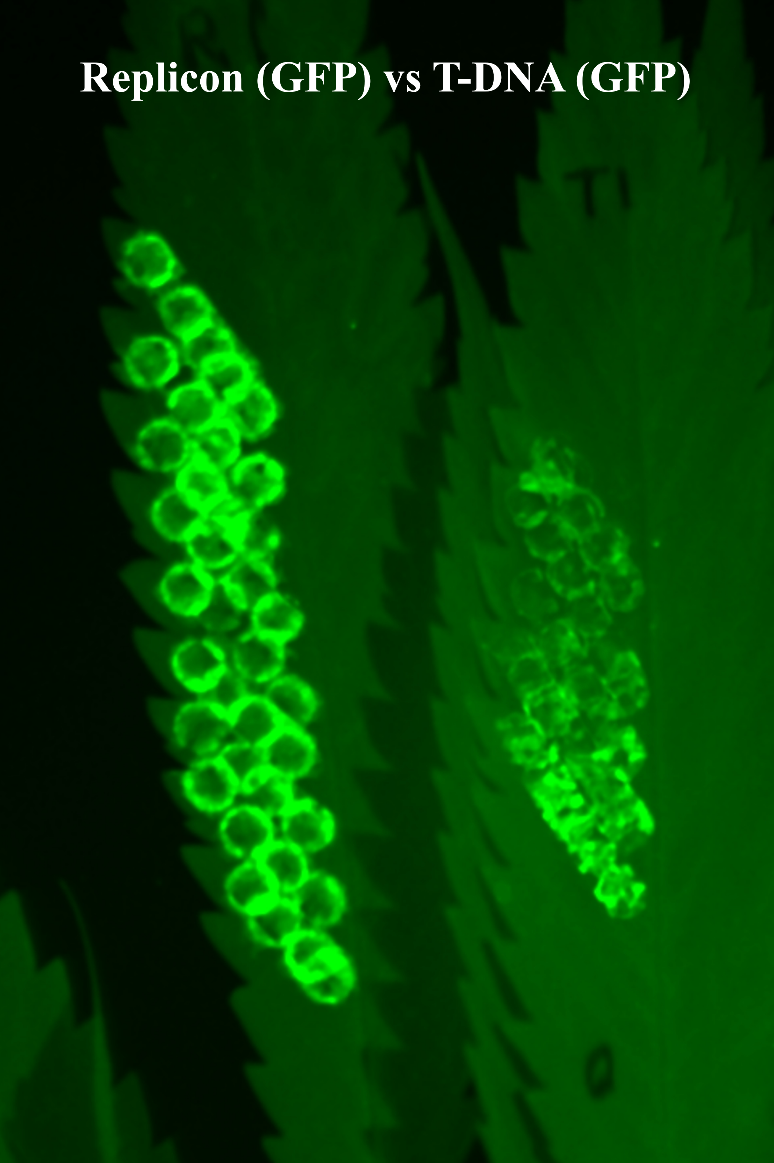


**Figure S4. Comparison of replicon and conventional binary vector using transient expression in *Cannabis sativa* leaves*.*** These plants were incubated for 4 days after agro-infiltration using *Agrobacterium* strains EHA105 carrying pLSL.R.Ly-GFP and pAGM4723-GFP, respectively.


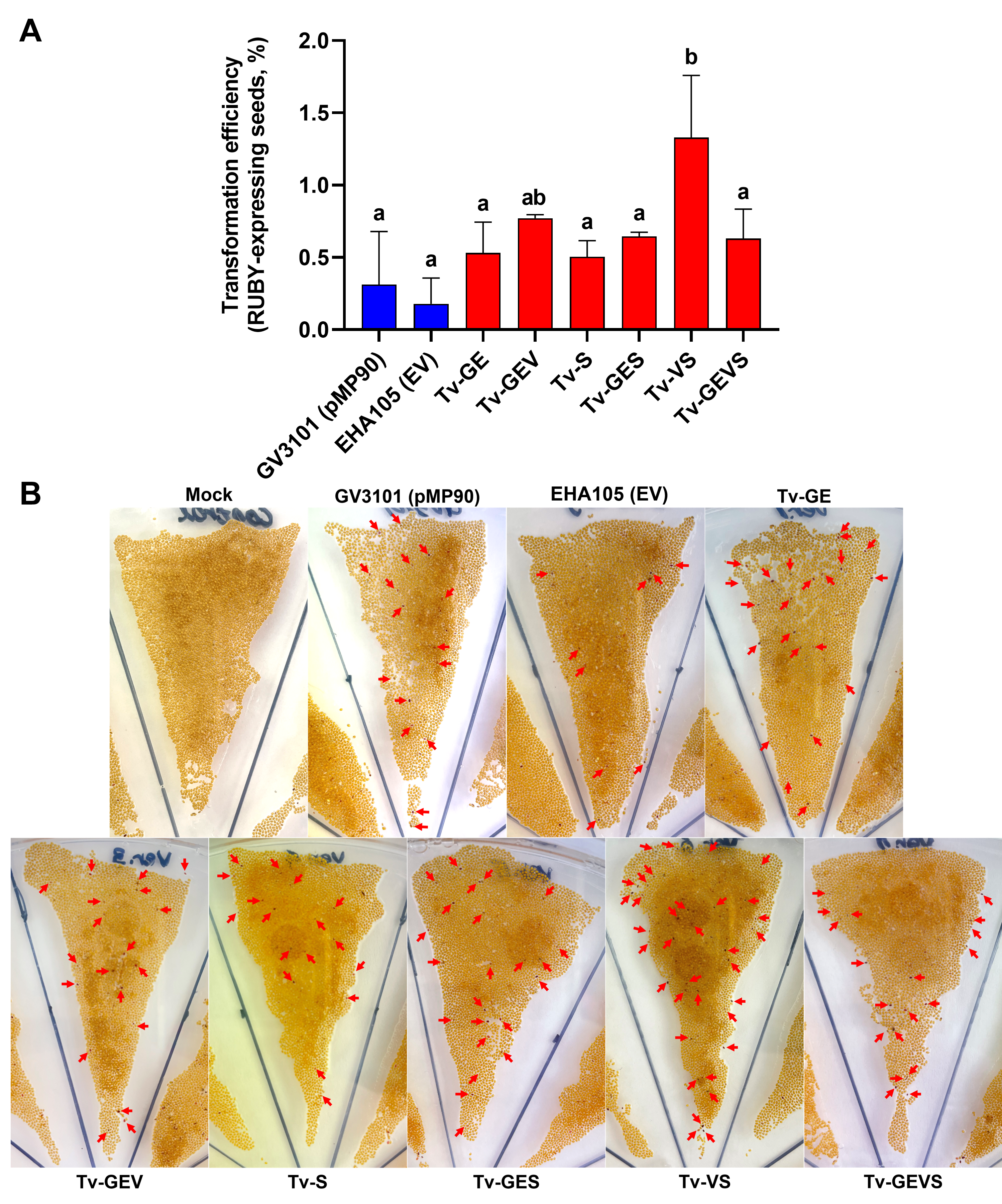


**Figure S5. *Arabidopsis* transformation with *Agrobacterium* strains carrying the visible RUBY reporter using the floral dipping method**. **(A)** Effect of Tv system on transformation efficiency in *Arabidopsis thaliana*. **(B)** RUBY-expressing transgenic seeds, which displayed dark red or pink color, were easily distinguishable in harvested seeds. Red arrows indicate RUBY-expressing seeds. Values are means ± SD (*n* = 3). Different characters indicate a statistically significant difference based on one-way ANOVA and Tukey’s multiple range test, with *P* < 0.05. *Agrobacterium* strains contained *RUBY*-construct.


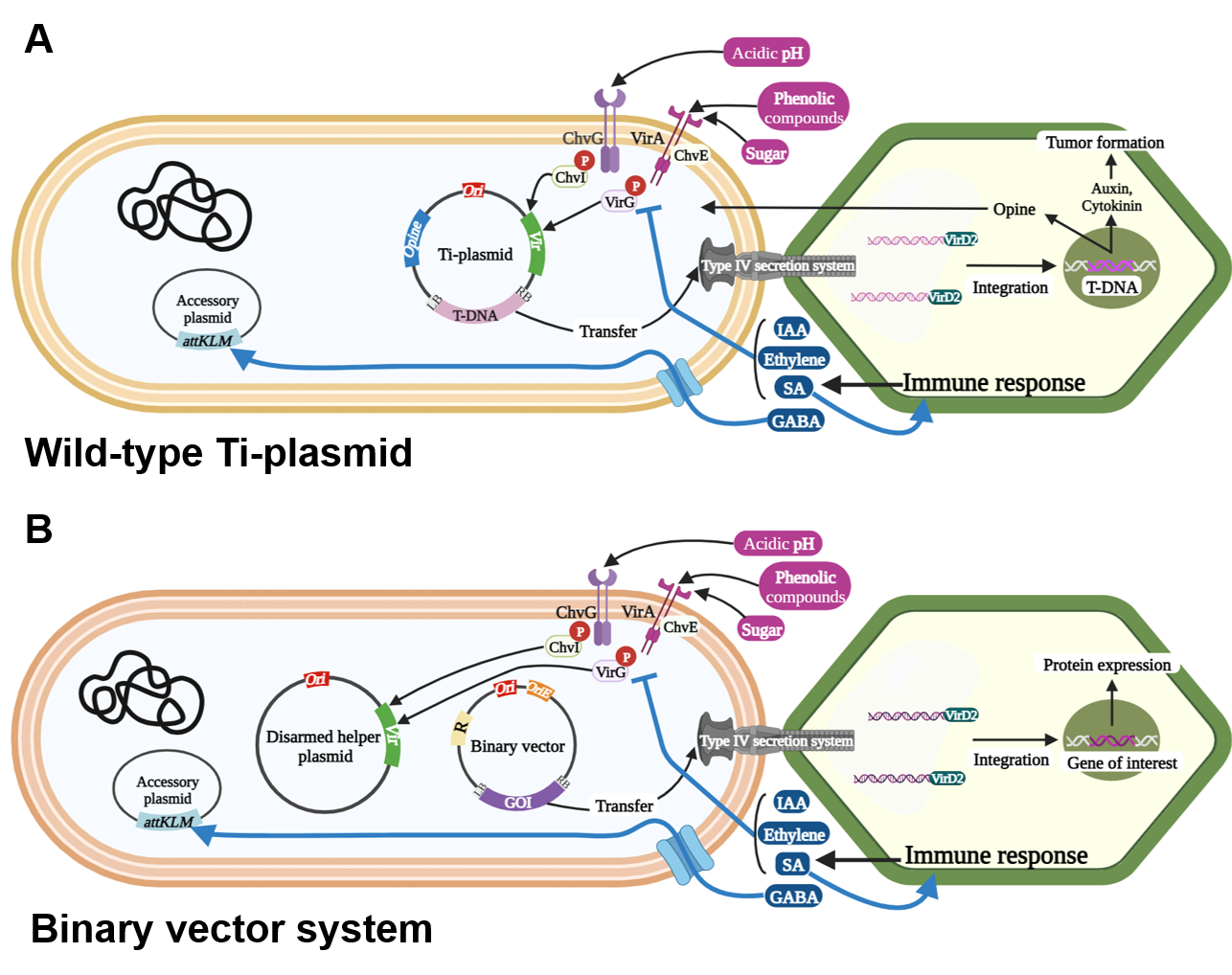


**Figure S6. Schematic representation of the development of *Agrobacterium*-mediated plant transformation.** **(A)** *Agrobacterium*-mediated T-DNA transfer process in wild-type *Agrobacterium* with Ti-plasmid. **(B)** In a binary vector system, Ti-plasmid has developed into helper plasmid and binary plasmid, facilitating the cloning of the genes of interest.


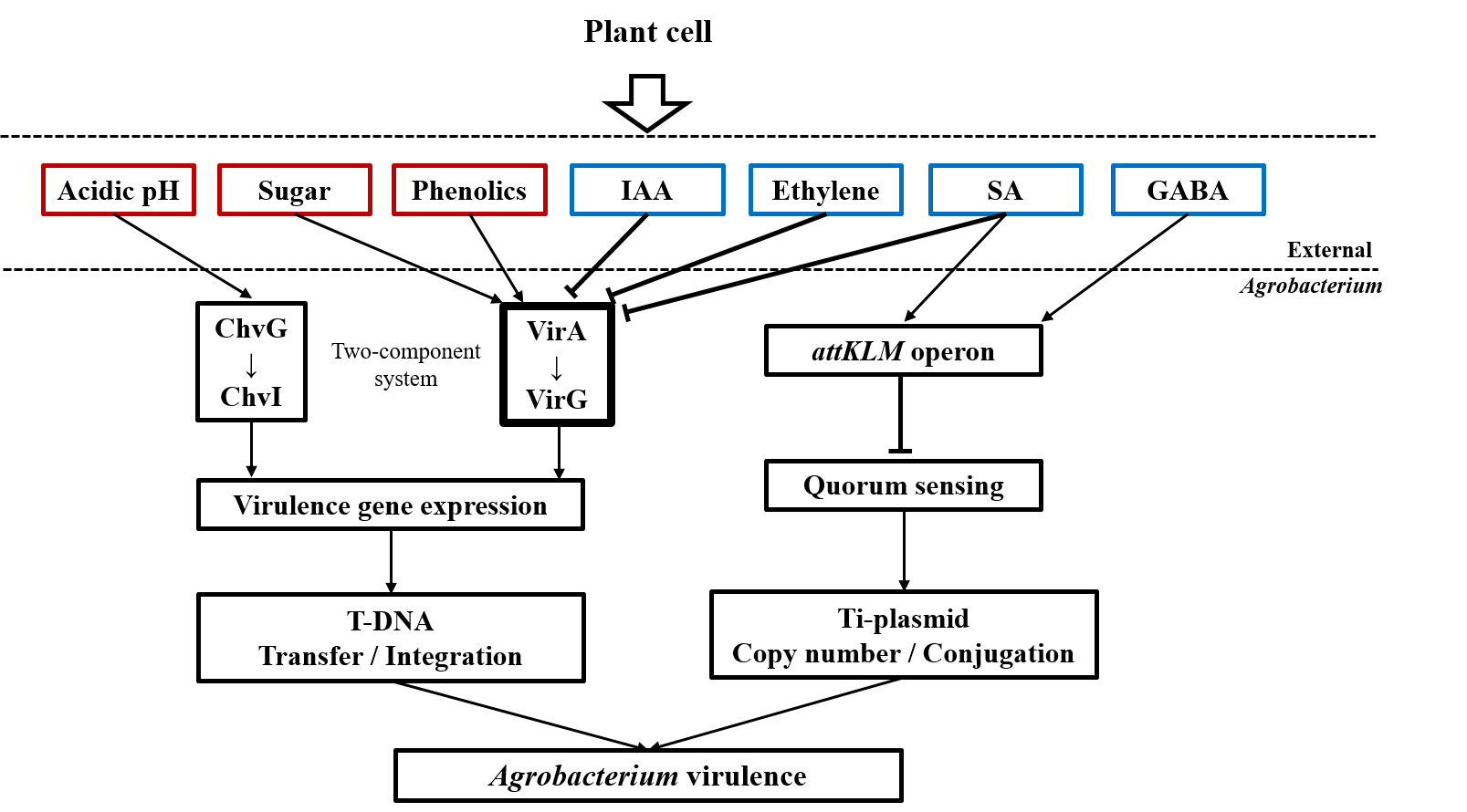


**Figure S7. Crosstalk between *Agrobacterium* virulence program and plant-derived signals.** VirA/VirG two-component system mainly modulates *Agrobacterium* virulence signaling through virulence gene induction. Another two-component system, ChvE/ChvI, also involves in this signaling. Whereas induction of the *attKLM* operon leads to the inactivation of quorum-sensing signals, thereby reducing the *Agrobacterium* virulence. Red boxes indicate positive molecules, blue boxes indicate negative molecules, the arrows indicate up-regulation, and the stopped arrows indicate down-regulation.
